# Identifying the Rules of Engagement Enabling Leukocyte Rolling, Activation, and Adhesion

**DOI:** 10.1371/journal.pcbi.1000681

**Published:** 2010-02-19

**Authors:** Jonathan Tang, C. Anthony Hunt

**Affiliations:** 1 UCSF/UC Berkeley Joint Graduate Group in Bioengineering, University of California, Berkeley, Berkeley, California, United States of America; 2 Department of Bioengineering and Therapeutic Sciences, University of California, San Francisco, San Francisco, California, United States of America; University of California San Diego, United States of America

## Abstract

The LFA-1 integrin plays a pivotal role in sustained leukocyte adhesion to the endothelial surface, which is a precondition for leukocyte recruitment into inflammation sites. Strong correlative evidence implicates LFA-1 clustering as being essential for sustained adhesion, and it may also facilitate rebinding events with its ligand ICAM-1. We cannot challenge those hypotheses directly because it is infeasible to measure either process during leukocyte adhesion following rolling. The alternative approach undertaken was to challenge the hypothesized mechanisms by experimenting on validated, working counterparts: simulations in which diffusible, LFA1 objects on the surfaces of quasi-autonomous leukocytes interact with simulated, diffusible, ICAM1 objects on endothelial surfaces during simulated adhesion following rolling. We used object-oriented, agent-based methods to build and execute multi-level, multi-attribute analogues of leukocytes and endothelial surfaces. Validation was achieved across different experimental conditions, in vitro, ex vivo, and in vivo, at both the individual cell and population levels. Because those mechanisms exhibit all of the characteristics of biological mechanisms, they can stand as a concrete, working theory about detailed events occurring at the leukocyte–surface interface during leukocyte rolling and adhesion experiments. We challenged mechanistic hypotheses by conducting experiments in which the consequences of multiple mechanistic events were tracked. We quantified rebinding events between individual components under different conditions, and the role of LFA1 clustering in sustaining leukocyte–surface adhesion and in improving adhesion efficiency. Early during simulations ICAM1 rebinding (to LFA1) but not LFA1 rebinding (to ICAM1) was enhanced by clustering. Later, clustering caused both types of rebinding events to increase. We discovered that clustering was not necessary to achieve adhesion as long as LFA1 and ICAM1 object densities were above a critical level. Importantly, at low densities LFA1 clustering enabled improved efficiency: adhesion exhibited measurable, cell level positive cooperativity.

## Author Summary

To gain access to sites of inflammation, leukocytes must first adhere to the blood vessel wall using integrin molecules. It has been hypothesized that integrin clustering is essential for sustaining adhesion prior to transmigration into the inflamed tissue. We cannot challenge such hypotheses directly because it is infeasible to measure molecular level events during the leukocyte adhesion process. At best correlative relationships have been made. The alternative approach undertaken was to experimentally challenge the hypothesized mechanisms in silico. We used object-oriented, software engineering methods to build and execute multi-level, multi-attribute analogues of leukocytes and binding surfaces. The simulated leukocytes contained diffusible objects (representing integrins) on their surface that were allowed to interact with binding partners on simulated endothelial surfaces. Validation was achieved across different experimental conditions, in vitro, ex vivo, and in vivo, at both the individual cell and population levels. Consequently, the finalized virtual mechanisms stand as a concrete, working theory about detailed events occurring at the leukocyte-surface interface during adhesion. We challenged mechanistic hypotheses by conducting experiments in which the consequences of multiple mechanistic events were tracked. We discovered that integrin clustering was not necessary to achieve adhesion as long as integrin and binding partner object densities were above a critical level. Importantly, at low densities integrin clustering enabled adhesion that exhibited measurable, cell level positive cooperativity.

## Introduction

Integrin-mediated adhesion is a crucial regulator of cell-cell interactions as well as cell migration, yet its causal mechanisms are still not fully understood. Improved biological insight into how surface component interactions control the distinct steps for leukocyte trafficking, beginning with rolling and adhesion, will enable us to begin precisely manipulating integrin-mediated adhesive interactions between cells [1]. Sustained leukocyte adhesion sufficient to resist blood flow shear forces is a precondition to subsequent trafficking events. There is strong correlative evidence that some degree of LFA-1 integrin clustering as observed in vitro through fluorescence microscopy is essential for efficient, sustained adhesion [2]–[4]. Moreover, LFA-1 clustering may facilitate rebinding events, enhancing leukocyte adhesion even further [5],[6].

The use of advanced microscopy techniques have greatly enhanced our understanding of leukocyte trafficking in various tissues by allowing for the visualization of this process at the level of capillaries [7]–[10]. However, even with current, state-of-the-art wet-lab and intravital microscopy methods, we cannot directly challenge those hypotheses (mentioned above) experimentally because it is infeasible to directly measure molecular level events during the leukocyte adhesion process. By employing relatively new, object-oriented, discrete event simulation methods in which quasi-autonomous components interact within and between nested biomimetic structures, it is feasible to construct, validate, and challenge concrete, working simulations of conceptualized mechanisms. The approach, methods, and objectives are different from those of the familiar inductive approach to modeling and simulation [11]. Upon discovering a system that validated under a variety of experimental conditions, we designed experiments that traced the consequences of specific mechanistic events and thus were able to test mechanistic hypotheses directly. We quantified the role of LFA-1 clustering in leukocyte–surface adhesion, and measured improvements in adhesion efficiency when they occurred. In addition, we determined if and when rebinding events could contribute to sustained adhesion.

To obtain those new mechanistic insights, we needed to create new, multi-level, multi-scale simulation capabilities that could be merged seamlessly with previously developed single cell methods [12]. Object-oriented, software engineering methods were used to implement and iteratively revise biomimetic rolling and adhesion mechanisms [11],[12]. The products of the process were many, extant (actually existing, working, observable) hypothetical mechanisms: these components assembled as specified, will produce observable multi-level mechanisms upon execution that are analogous to those hypothesized to be responsible for leukocyte rolling, adhesion, and sustained adhesion. The objects included leukocyte analogues having multi-level surface components including diffusible LFA-1 analogues capable of clustering. We followed a rigorous, iterative refinement protocol to shrink the set of plausible biomimetic (biologically emulated) mechanisms. Simulation of finalized mechanisms led to validation: multiple, diverse, phenomena similarities met prespecified similarity criteria for over a dozen different wet-lab experimental conditions, in vitro, ex vivo, and in vivo at both the cell and population levels. Consequently, the mechanisms presented herein stand as a cohesive, concrete, tested and challengeable, working theory about detailed events occurring at the leukocyte–surface interface during rolling and adhesion experiments.

Our simulations enabled us to view how different molecular interaction events on simulated leukocyte surfaces cause behaviors that are unique and diverse at both the molecular and leukocyte levels, and yet—importantly—narrowly constrained at the population level. Within the validated system, clustering of LFA1 objects was not necessary to achieve adhesion as long as LFA1 and ICAM1 object densities were above a critical level. However, when LFA1 and ICAM1 object densities were both low and LFA1 clustering was enabled, adhesion between leukocyte and simulated endothelial surface exhibited measurable, positive cooperativity. No such cooperativity was evident when densities were high.

Early during simulations, there were no differences in LFA1 rebinding events with or without having LFA1 clustering enabled. Thereafter, however, clustering caused LFA1 rebinding (to ICAM1) to increase. The situation was somewhat different for ICAM1 rebinding. As early as 25 seconds into an experiment, significantly more ICAM1 rebinding (to LFA1) was measured when LFA1 clustering was enabled.

Class IB phosphoinositide-3 kinase (PI3Kγ) signal transduction molecules play important roles in regulating inflammation. Recruitment of leukocytes lacking PI3Kγ is impaired in several animal models of inflammation. However, details of how PI3Kγ regulates recruitment have not been determined [13],[14],[15]. PI3Kγ is upstream of many signaling pathways and is responsible for many additional leukocyte functions including chemotaxis, secretion, and the neutrophil respiratory burst [16]. To study the role of PI3Kγ in neutrophil adhesion, Smith et al. compared the behaviors of neutrophils from PI3K knockout (KO; PI3Kγ^−/−^) and wild-type (WT) mice in ex vivo flow chambers coated with P-selectin (substrate for rolling), ICAM-1 (substrate for adhesion), and CXCL1 (arrest chemokine for leukocyte activation and subsequent induction of PI3Kγ signaling). Neutrophils from KO mice, compared to WT counterparts, had a reduced ability to adhere to substrate-coated surfaces. Similar experiments were undertaken in vivo in exteriorized cremaster muscle venules of mice injected with CXCL1, and the results were similar. Neutrophils from KO mice, compared to WT counterparts, adhered less to the CXCL1-treated venular surfaces. Interestingly, most neutrophils from KO mice that were able to initiate adhesion were only able to do so for short intervals [13]. They hypothesized that the defects in adhesion observed ex vivo and in vivo were a result of an inability of LFA-1 to redistribute and cluster on the membrane of leukocytes in the PI3Kγ KO mice [13]. We used simulation experiments to directly test this hypothesis in silico. LFA1 objects were designed to cluster upon multivalent binding to multivalent ICAM1 objects, in an analogous fashion to what was observed in vitro by Kim et al [2]. Inhibiting this LFA1 clustering mechanism in silico caused simulated neutrophil behaviors that closely mimicked the above adhesion defect results from both ex vivo and in vivo experiments in KO mice across eight different experimental conditions at both the cell- and population-level, including the transient adhesion of simulated KO neutrophils. Thus, the neutrophil–surface interaction model presented herein stands as a tested theory about the mechanistic events that may be occurring in normal and KO mice during neutrophil rolling and adhesion. Experimenting on descendants of these models may help speed discovery and development of critically needed immunotherapeutics [17].

### Biological Background

Rolling, activation, and adhesion are critical events in the inflammatory response, as they are required for the proper recruitment of leukocytes to sites of tissue damage. The selectin family of receptors and their respective carbohydrate ligands largely mediate the initial interactions between leukocytes and endothelial cells. Their high frequency rates of association and dissociation allow for transient interactions that slow the speed of travel, enabling leukocytes to roll along the activated endothelial surface and integrate inflammatory signals, such as immobilized chemokines [18].

The transition from rolling to adhesion is exclusively mediated by integrin receptors. They can exist in multiple conformational states each having different ligand binding properties. Low-, intermediate-, and high-affinity states have been identified. Natively, these integrins exist in non-adhesive, low-affinity states to prevent leukocytes from sticking non-specifically to blood vessel surfaces. Upon detection of immobilized chemokines, such as CXCL1, intracellular signaling events can trigger integrin conformational changes to high affinity states that enable a leukocyte to adhere firmly [19]. Post-adhesion events involving integrins are thought to help strengthen the attachment to the endothelium [13],[20].

Earlier studies, in addition to those cited in the Introduction, have provided evidence that LFA-1 clustering is mediated by PI3K and that the process is important for leukocyte adhesion. Constantin et al. showed that chemokines triggered a rapid increase in LFA-1 affinity on lymphocytes. Using immunofluorescently labeled LFA-1 with confocal microscopy, they observed that chemokines also stimulated LFA-1 movement into clusters and large polar patches [4]. Inhibiting PI3K activity blocked LFA-1 mobility but had no effect on LFA-1 affinity change. In separate experiments, PI3K inhibitors prevented lymphocytes from adhering to low densities of immobilized ICAM-1 substrate. At high ICAM-1 densities, inhibiting PI3K had no effect on lymphocyte adhesion. Whether this same phenomena exists in neutrophils remains to be determined.

## Methods

### Executable Biology Model

**Figure 1:**
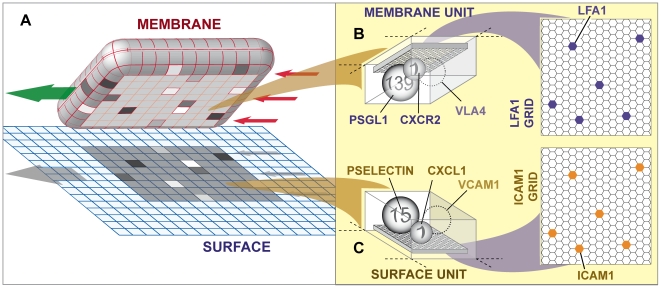
Sketch of the ISWBC2 experimental system components. (A) A leukocyte agent is shown pulled away from the simulated surface to which it was attached. The left arrow indicates roll direction; the three right arrows indicate shear resulting from the simulated flow. The surface is discretized into independent units of function called surface units. The leukocyte's membrane is similarly discretized into matching units called membrane units: 600 total (20×30). The 8×10 shaded region on the surface and on the leukocyte's underside identifies the contact zone. The units within the contact zones that are shaded differently indicate that different numbers of bonds had formed between ligand–ligand pairs in overlapping units; otherwise, no bonds formed. Rolling is the result of a sequence of forward ratchet events. One ratchet event is the result of one row of membrane units being released at the rear of the contact zone along with engagement of a new row of at the front of the contact zone. One ratchet event maps to a leukocyte rolling approximately 1 µm (relative to the flow chamber surface). (B) A membrane unit is illustrated. Each is a software object functioning as a container. All leukocyte membrane functionality (relevant to these studies) within each unit of surface on icam1Grid is represented by the four objects: psgl1, vla4 (not used for most experiments), cxcr2, and lfa1. They map to leukocyte receptors; they are illustrated as spheres. The number on each sphere indicates the number of receptors to which that agent maps. Mobile lfa1 objects reside on a lfa1Grid. Each lfa1 maps to an individual LFA-1 molecule. (C) A surface unit is illustrated. Similar to membrane units, each surface unit is simulated using a software object functioning as a container. All flow chamber surface functionality (relevant to these studies) within each surface unit is represented by four objects: pselectin, vcam1 (not used for most experiments), cxcl1, and icam1. They map to surface receptors and chemokines. The number on each sphere indicates the number of receptors or chemokines to which that agent maps. Similar to lfa1, an icam1 resides on icam1Grid, a 2D hexagonal lattice, and maps to an individual ICAM-1 molecule.

The simulation system we have constructed is designed to be an experimentally useful analogue of the wet-lab experimental systems used to study leukocyte rolling and adhesion (Figure 1). Wet-lab experimental systems provide data that can be used to generate mechanistic hypotheses. We used the synthetic modeling method, an example of what has been referred to as executable biology [21],[22], as a means to instantiate those mechanistic hypotheses so that they can be evaluated and tested. Object-oriented, agent-based software components were designed, instantiated, verified, plugged together, and then operated in ways that can map concretely to mechanisms and processes believed responsible for leukocyte rolling and adhesion. An agent is a quasi-autonomous object capable of scheduling its own actions in much the same way as we imagine cells and some of their components (such as a mitochondrion or modular subsystem) doing. See [12] for details of this approach, the basic framework of the in silico white blood cell (ISWBC), and an iterative refinement protocol for successively developing and validating simulation results. To distinguish the previous version from the current one, we refer to the former as ISWBC1 (in silico white blood cell) and the latter as ISWBC2. Below we summarize details and the new ISWBC2 capabilities.

### Iterative Refinement Method

The greater the similarity between the measured behaviors, or phenotype, of an ISWBC2 and corresponding wet-lab attributes of interest, the more useful it will become as a research tool and as an expression of the coalesced, relevant leukocyte knowledge. The expectation has been that increasing phenotype similarity will require, and can be achieved in part through, similarities in design plan and in generative mechanisms. We follow an iterative refinement protocol to systematically and sequentially extend the overlap of the model and referent system phenotypes. This iterative refinement protocol allows us to concatenate in a dynamic fashion new knowledge about the referent systems into the synthetic model, without having to reengineer the whole system, and without having to compromise already validated features and behaviors [12], [23]–[25]. This approach has its grounding in the use of modeling and simulation as a research topic [26]. It is as follows:

- For the wet-lab systems being studied, create a list of attributes to be targeted.
- Design, construct, and enable the analogue to exhibit the targeted phenotypic attributes.
- Iteratively falsify and revise the operating mechanisms until the analogue exhibits the targeted phenotypic attributes within a pre-specified level of similarity, thereby achieving a level of validation.
- The process is then repeated with the addition of more phenotypic attributes to the targeted list.

It is important to note that validity is distinguishable from verity. For this technical context, we define validity as the degree to which an assertion (such as an ISWBC2) can be trusted as true enough or true to within some tolerance (e.g., the similarities discussed herein). In contrast, verity targets ontological truth, regardless of any arguments or belief. One cannot verify a representation such as an ISWBC2 against reality because of the ontological wall. Therefore, we define (http://biosystems.ucsf.edu/research_dictionary.html) verification as the process of determining where two statements agree and disagree, as in comparing a model to its corresponding simulation. One can only validate a representation against reality. Model validation can be achieved at several levels [27]. For example, one level of validation is through the qualitative reproduction of observed behaviors. Another level can be determined through parameter variability and sensitivity analysis. Changes to the values of input and internal parameters should affect model behaviors in a fashion similar to when analogous changes are made to the real system. Additionally, a level of validation can be achieved through the reliable prediction of new data. The most appropriate type and level of validation will depend on the intended model usage. ISWBCs were designed specifically to explain the diverse observations of leukocytes as they interact with endothelial surfaces, which is in contrast to inductive mathematical models that are typically intended for precise prediction [11].

We previously reported progress validating against our initial set of targeted attributes [12]. The ISWBC1 successfully represented the dynamics of individual leukocytes rolling separately on P-selectin and VCAM-1, along with the transition from rolling to adhesion on P-selectin and VCAM-1 in the presence of GRO-α chemokine. Additionally, the individual in silico and in vitro behavioral similarities translated successfully to population-level measures (Table 1A). Herein we extend the model by also targeting the phenotypic attributes of LFA-1 and ICAM-1 receptor mobility and clustering (Table 1B). Doing so allowed us to observe their hypothesized role in initiating adhesion ex vivo and sustaining adhesion in vivo.

**Table 1:** Targetable phenotypic attributes of leukocytes in vitro/ex vivo/in vivo: an abridged list.

Phenotypic Attribute	Reference
*Set A: previously targeted attributes*	
Characteristic jerky stop and go movement during rolling	[12],[50]
Highly fluctuating rolling velocities	[12],[51]
Larger rolling velocities observed at higher shear rates	[12],[52]
Smaller rolling velocities at higher ligand substrate densities	[12],[52]
Rolling a velocities on pselectin a match reported values	[12],[52]
Small number of bonds within the contact zone, e.g., within 2–20	[12],[53]
Distance-time and velocity-time data for rolling a on a pselectin/vcam1 are indistinguishable from reported data	[12],[50],[51]
Chemokines induce adhesion within seconds	[12],[54]
*Set B: currently targeted attributes*	
LFA-1 and ICAM-1 lateral mobility and diffusion	[55]
LFA-1 nanocluster formation upon binding multivalent ligand	[2]
ICAM-1 spatial configurations in vivo	[33]–[37],[56]
Effect of phosphoinositide 3-kinase inhibitors on adhesion ex vivo	[13]
Effect of phosphoinositide 3-kinase inhibitors on adhesion in vivo	[13]
*Set C: future targetable attributes*	
Induction of LFA-1-dependent neutrophil rolling on ICAM-1 by engagement of E-selectin	[57]
Synergistic effect observed during neutrophil rolling on P- and E-selectin	[58]
Effect of knocking out VAV1/3 guanine nucleotide exchange factors on neutrophil rolling	[20]
Effect of inhibitors to other signaling molecules on cell arrest (pertussis toxin (PTx)-sensitive G proteins, p38 mitogen-activated protein kinase)	[57]

We find that this model accounts for the nonlinear influence of experimentally induced visual motion on human postural behavior both in our data and in previously published results.

a We use small caps when referring to the in silico components, features, measurements, and events.

### The Leukocyte Analogue

We used RePast 3 as our modeling and simulation framework. It is a java-based software toolkit developed at the University of Chicago for creating and exercising agent-based models (http://repast.sourceforge.net/repast_3/index.html). The libraries provided were used to create, run, display, and collect data. The ISWBC2 can be downloaded at [28].

To avoid confusion and clearly distinguish model components, features, measurements, and events from their *ex vivo* or *in vivo* counterparts, we use small caps when referring to those of analogues. The biological aspects of the referent experimental systems and their ISWBC2 counterparts are listed in Table 2.

**Table 2:** Table of biological aspects from the experimental system and their ISWBC2 counterparts.

Biological Aspects	Model Components	Description or Type
Substrate-Coated Surface	Surface	2D Square Lattice (low resolution)
Functional Unit of the Surface	Surface Unit	Grid Unit of the Surface
Surface area containing ICAM-1	icam1grid	2D Hexagonal Lattice (high resolution)
Leukocyte	Leukocyte	Object
Leukocyte Membrane	Membrane	2D Square Lattice (low resolution)
Functional Unit of Leukocyte Membrane	Membrane Unit	Grid Unit of the Membrane
Membrane area containing LFA-1	lfa1grid	2D Hexagonal Lattice (high resolution)
Chemokine	Cxcl1	Object
Chemokine Receptor	Cxcr2	Receptor Object
Adhesion Molecule	Adhesion Molecule	Receptor Object
Tensile Force on Rear Bonds	Parameter: *RearForce*	Parameter representing force due to shear
Hypothesized Biological Mechanisms	Operating Mechanisms	Algorithms

Surface maps to either the endothelial cell surface or flow chamber surface depending on the simulation experiment.

The surface with which leukocytes interact is discretized into independent units of function called surface units. The leukocyte's membrane is similarly discretized into matching units of function called membrane units. For simplicity, the surface and membrane are both implemented as 2D toroidal lattices. With current parameter values, one surface unit maps to approximately 1 µm^2^ and when rolling or adhered, one membrane unit maps to the same amount of surface area on the cell membrane. Contained within each unit are receptor objects, each one mapping to receptors found on the surface or leukocyte membrane. An eight × ten unit region shared between the surface and the membrane identifies the contact zone. It determines which surface and membrane units (and which of their receptors) can interact.

In ISWBC1s [12], the receptors found at the tips of microvilli (PSGL-1, VLA-4, and CXCR-2) were represented by the receptor objects psgl1, vla4, and cxcr2. Each one mapped to several binding molecules of the same type that may be found within a discrete area within the referent system. For example, a psgl1 receptor mapped to several PSGL-1 adhesion molecules. The approximate number to which that object maps is determined by its parameter, *TotalNumber*. In ISWBC2, we have increased granularity (spatial resolution) by adding receptor objects lfa1 and icam1 and placing them within a higher granularity 2D hexagonal grid (lfa1grid and icam1grid) within some membrane units and surface units, respectively, as illustrated in Figures 1B,C. lfa1 maps to the integrin molecule LFA-1 that is found on leukocyte membranes between microvilli [29],[30], while icam1 maps to its ligand-receptor ICAM-1. Distinct from the other receptor objects, each lfa1 and icam1 object maps to single molecules. Each high-resolution hexagonal grid space maps to approximately 100 nm^2^ of leukocyte membrane or surface.

### Behaviors

ISWBC2 experiments are analogous to those performed in vivo or using an ex vivo flow chamber system. While on the surface, leukocytes use their receptors and the decisional processes sketched in Text S1 to interact and form bonds with receptors on the surface. Those interactions are recorded and measured. The ISWBC2 consists of components having three levels of spatial resolution illustrated in Figure 1: Leukocyte-level, Membrane/Surface Unit-level, and lfa1 grid/Icam1 grid-level. High-level behaviors are dependent upon the collective operation of objects and agents contained within each of the lower levels. For example, the behavior of membrane and surface units arise from the receptor objects contained within each. Similarly, the behavior at the leukocyte-level is dependent upon the collective events that occur within the underlying membrane/surface units. Conversely, events at the highest level impose constraints upon allowed lower level behaviors. For example, the positioning and movement of the leukocyte on the surface dictate which membrane and surface units are overlapping and can interact.

### Receptor Behaviors

Local activation of an integrin within a membrane unit occurs when a chemokine receptor detects a chemokine in an overlapping surface unit. When an activation signal is detected, local lfa1 receptors change from low to high affinity state. They achieve that by changing the parameter values *Pon* (bond formation rates), bond b_0_, b_1_, and b_Limit_ (bond dissociation properties), as described in Text S1. Relationships between the parameter *bondforce* and probability of bond dissociation for each of the four ligand pairs included in the ISWBC2 are given in Text S1 along with corresponding in vitro measurements. The diffusive properties of lfa1, specified by the parameter *LFA1MoveNum*, are also dependent on state. Lfa1 lateral mobility parameters were specified as described Text S1 such that they have diffusive properties similar to those observed in vitro. Values are provided in Text S1. *LFA1MoveNum* determines the number of attempts that an lfa1 object will take to move to a neighboring space within the lfa1grid during a single simulation cycle. During lateral movement, Lfa1 has an equal probability of moving into any of its six neighboring spaces, but it cannot move into an already occupied space. When simulating the endothelial cell surface, icam1 can similarly diffuse on the icam1grid with a rate determined by the parameter *ICAM1MoveNum*. As a simple representation of some LFA-1 trafficking events (removal from endocytosis, deactivation, extraction from the membrane) [31],[32], unbound lfa1 is removed from the membrane during each simulation cycle with a probability of *LFA1RemovalRate*.

### Leukocyte Movement

The number and location of bonds at the surface and membrane unit level combined with the decisional processes sketched in Text S1 determine Leukocyte behavior. If there are bonds between adhesion molecules within the rear column of the contact zone, the leukocyte pauses, or remains stationary, until the next simulation cycle. If there are no bonds within the rear column of the contact zone, the leukocyte, influenced by the simulated shear force, performs a forward rolling movement. Rolling is the result of a sequence of forward ratchet events. The process involves removing a column from the rear of the membrane's rectangular contact zone while a new one is placed at the front of the zone above the surface.

### Experimental Behaviors and Similarity Modeling Approach and Strategy

At the beginning of each simulation experiment, Boolean parameters (Table 3) determine whether Lfa1 clustering (*LFA1Clustering*), preformed icam1 clustering in endothelial surface units (*ICAM1Pre-Clustered*), or icam1 tetramer formation (*ICAM1Tetramer*) is allowed to occur.

**Table 3:** Boolean variables determining which rules and behaviors are allowed during a simulation.

Boolean Parameter Name	Description
*LFA1Clustering*	Allow LFA1 agents to cluster?
*ICAM1Pre-Clustered*	Allow ICAM1 agents to be prearranged into cluster?
*ICAM1Tetramers*	Allow ICAM1 agents to form tetramers upon ligand-binding?

When mean ISWBC2 results were within the range of mean ± SD of wet-lab results, the two sets of data were declared experimentally indistinguishable.

### Lfa1 and icam1 clustering, and tetramers

**Figure 2:**
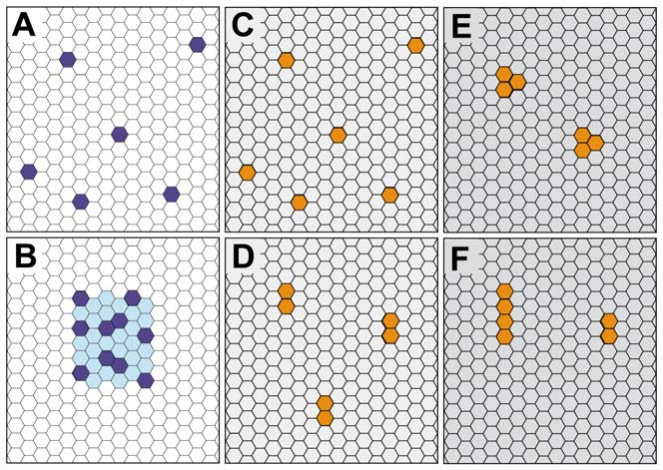
Diagrams illustrate the different spatial configurations of lfa1 and icam1 adhesion molecules implemented and tested during simulations. lfa1Grids are shown with lfa1 objects labeled dark blue in either (A) a nonclustered state or (B) a clustered state. Clustered lfa1 are spatially restricted within the light blue region. Icam1Grids are shown with icam1 objects labeled yellow in either a (C) native monomeric state, (D) a native dimeric state, (E) as preformed clusters, or (F) as w-tetramers formed after ligand-binding.

A lfa1 cluster on the lfa1Grid is specified by the parameter *LFA1ClusterDiameter*. It determines the length and width of the region that LFA1 objects must stay within while diffusing on the lfa1grid. Lfa1 clusters are formed by randomly choosing non-overlapping regions on the lfa1grid and then filling each with all Lfa1 within that membrane unit (Figure 2B). The number of clusters per lfa1grid is specified by the parameter *NumLFA1Clusters*. During a simulation, clusters with unbound lfa1 randomly move to new and unoccupied locations within the lfa1grid, a process that maps to molecular diffusion within a membrane region.

Different configurations of ICAM-1 have been reported in the literature. One study suggested that the native state of ICAM-1 is monomeric, because a monomer contains the complete binding site for LFA-1 [33]. Other studies provided evidence that dimeric ICAM-1 is the predominant form expressed by cytokine-activated endothelium [34],[35]. One such study showed that a dramatic conformational change of dimeric ICAM-1 occurs upon binding to LFA-1 such that they form one-dimensional chains of W-tetramers and higher order oligomers [36].

However, more recent studies by Barreiro et al. observed that ICAM-1 is clustered with other adhesion molecules into preformed tetraspanin-enriched microdomains on the surface of activated endothelial cells. They provided convincing evidence that these preformed microdomains might act as specialized endothelial adhesive platforms for leukocytes during adhesion and extravasation [37]. Evidence from immuno-electron microscopy of fixed endothelial cells suggested that these organized microdomains might be smaller than 100 nm in diameter [38]. Scanning electron microscope images of activated endothelial cells stained with anti–ICAM-1 antibodies followed by 40-nm gold immunolabeling showed a cluster size of 2.4±0.1 (mean ± SE) particles per nanoclusters.

**Figure 3:**
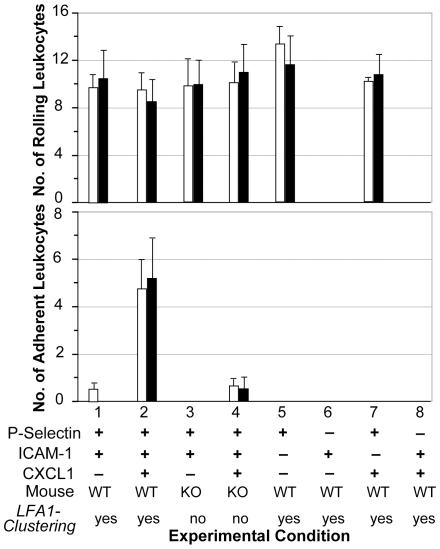
Ex vivo and in silico results for eight different experimental conditions are compared. Ex vivo conditions (from [13]): the flow chamber surface was coated with P-selectin and/or ICAM-1 with or without immobilized CXCL1 chemokine. Mice were either WT or PI3Kγ knockouts (KO). Leukocytes that rolled and adhered within each of five fields of view were recorded during a 60-second observation interval. White bars are ex vivo means ± 1 SE. The paired black bars are ISWBC2 means ± 1 SD (20 populations containing 30 leukocytes each) for the same condition using the parameter values in Tables 4 and 5. ISWBC2 experiments that map to WT mouse counterparts used *LFA1Clustering* = true. Simulations of KO mice used the parameter setting *LFA1Clustering* = false.

In our simulations of in vivo experiments, we implemented the latter case where ICAM-1 exists preclustered (*ICAM1Pre-Clustered* = true). However, we also implemented the three other hypothesized ICAM-1 spatial configurations in order to observe their relative effects on leukocyte adhesion: (1) native monomeric ICAM1 (Figure 2C), (2) native dimeric ICAM1 (Figure 2D), and (3) in Figure 2F, dimeric ICAM-1 forming linear W-tetramers upon ligand binding (*ICAM1Tetramer* = true).

In the simulations, when *ICAM1Tetramer* = true, a bound icam1 forms a linear tetramer structure with a nearby unbound dimeric icam1 object (Figure 2F). To keep things simple in simulations where *ICAM1Pre-Clustered* = true, preformed ICAM-1 nanoclusters were represented using three icam1 molecules held together (Figure 2E).

### Sensitivity Analysis and rebinding

Upon achieving leukocyte behaviors that matched those from ex vivo and in vivo experiments, robustness to changes in a variety of key parameters were measured. Small (5–10%) and large (50%) parameter value changes were made to *Pon* (high affinity lfa1-icam1) (bond formation probabilities for lfa1-icam1), *RearForce*, and *LFA1RemovalRate*, while other factors were held constant. For each, ISWBC2 behaviors were recorded.

During a simulation, each lfa1 and icam1 object kept track of its bond event and position history. When needed, that information was transferred to a separate file. Each membrane unit and surface unit also kept track of the number of bond events that involved receptor objects contained within, including lfa1 and icam1 rebinding events.

## Results

### Re-validation studies of rolling on pselectin

Many iterative refinement cycles were performed in order to get from ISWBC1 to ISWBC2. While no code changes were made to the leukocyte receptors psgl1 and vla4 or to the substrates pselectin and vcam1, software changes were made to manage the simulation output. Therefore, we deemed it necessary to test the ISWBC2's ability to reproduce some of the essential targeted phenotypic attributes from Table 1 that were previously used to validate the ISWBC1.

We repeated simulations of in vitro parallel plate flow chamber experiments that observed neutrophils rolling on various densities of P-selectin (9 and 25 sites/µm^2^) and under varying wall shear rates (0.5, 1.0, and 2.0 dyn/cm^2^), exactly as executed previously [12]. Using analogous in silico experimental conditions, the ISWBC2 leukocytes produced behaviors (not shown) that were indistinguishable from wet-lab results and from simulation results previously reported. Leukocytes exhibited the characteristic jerky stop-and-go movement with highly fluctuating rolling velocities. Average rolling velocities were calculated and were still within the ranges reported in the literature. Higher average leukocyte rolling velocities were observed at higher simulated shear rates. At higher pselectin substrate densities, leukocytes rolled with smaller average rolling velocities. Lastly, comparison of pause time distributions revealed no apparent difference between the ISWBC1 and ISWBC2. From these studies, we concluded that the new code introduced to transform ISWBC1 into ISWBC2 did not affect the model's ability to reproduce previously targeted phenotypic attributes.

### Lfa1 clustering and leukocyte adhesion

To gain insight into the role of PI3Kγ on leukocyte rolling and adhesion under flow conditions, Smith et al. compared the behaviors of leukocytes from PI3Kγ KO and WT mice ex vivo using blood-perfused micro-flow chambers coated with the endothelial cell substrate molecules P-selectin, ICAM-1, and CXCL1 [13]. Nine one-minute recordings of a field of view were taken of the center of each chamber under each experimental condition. Population-level measures of rolling and adhesion were obtained by averaging the number of rolling and adherent cells for each condition. Arrested cells were defined as those that were adherent for at least 30 s. They observed a reduced ability of leukocytes from PI3Kγ KO mice to adhere to the coated surfaces in comparison to leukocytes from WT mice.

We simulated analogous experimental conditions to determine if the addition of a simple lfa1 clustering mechanism and its inhibition would enable the in silico system to mimic that adhesion defect. We used the same combination of substrate analogues. We explored the consequences of changing *LFA1Clustering* in isolation of other variables during each experiment. We chose parameter values based on reported literature values or searched empirically for parameter sets that would provide acceptable matches for all eight experimental conditions. Listed parameters found in the literature were from experiments specifically using murine neutrophils. The leukocyte parameters from Table 4 and the environment parameters from Table 5 (part II) are such a set; they produced the results in Figure 3. The data are averages from 20 sets of experiments containing 30 leukocytes each, with the duration of each run being 600 simulation cycles (maps to 1 minute). The number of rolling and adhering leukocytes for each batch were counted and averaged. Leukocytes that remained stationary on the surface for at least 300 simulation cycles (about 30 seconds) were classified adherent. Figure 3 shows that for all ligand combinations and genotypic variations simulated, both the leukocyte rolling and adhesion data matched that from ex vivo experiments: the in silico and wet-lab results were indistinguishable experimentally.

**Table 4:** Parameter values for the leukocyte membrane and ligands along with corresponding wet-lab values.

Parameter Name	Description	Model Parameter Value	Experimental Value	Reference
*LeukTotalWidth*	Leukocyte membrane width (in the *y* [east-west] dimension)	20 membrane units	Average murine Neutrophil Diameter: ∼7 µm	[59]
*LeukTotalLength*	Leukocyte membrane length (in the *x* [north-south] dimension)	30 membrane units	Average murine Neutrophil Diameter: ∼7 µm	[59]
*LeukExposedWidth*	Contact zone width (in the *y* dimension)	8 membrane units	NA	NA
*LeukExposedLength*	Contact zone length (in the *x* dimension)	10 membrane units	NA	NA
*LFA1GridSize ICAM1GridSize*	Length/width of lfa1Grid and icam1Grid	100×100	NA	NA
*LFA1GridDensity*	Density of lfa1Grid on membrane exposed to the surface.	0.20	NA	NA
*NumLFA1Clusters*	Number of lfa1 clusters/lfa1Grid formed if clustering is initiated	2	N/A	N/A
*LFA1ClusterDiameter*	The length and width of the lfa1 cluster on the lfa1Grid	6	N/A	N/A
*PSGL1Density*	Mean number of PSGL-1 molecules (± standard deviation) represented by each psgl1 agent	120±5	∼75,000/murine neutrophil^a^	[60]
*LFA1Density*	Mean number of LFA-1 molecules (± standard deviation) in each membrane unit	25±5	∼50,000/murine neutrophil^a^	[61]
*LFA1RemovalRate*	Probability that an unbound lfa1 will be removed from the membrane	0.0025	NA	[31],[32]
*CXCR2Density*	Number of CXCR-2 molecules (± standard deviation) represented by each cxcr2 agent	1	NA	NA
*Pon* (psgl1-pselectin)	Probability of forming a psgl1-pselectin bond	0.001	NA^b^	[62]
*Pon* (low affinity lfa1-icam)	Probability of forming a low affinity lfa1-icam1 bond	0.01	NA^b^	[63]
*Pon* (high affinity lfa1-icam1)	Probability of forming a high affinity lfa1-icam1 bond	1.0	NA^b^	[64],[65]
*Pon* (cxcr2-cxcl1)	Probability of cxcr2 interacting with cxcl1	1.0	NA	NA
*Poff* (cxcr2-cxcl1)	Probability of cxcr2 releasing cxcl1	1.0	NA	NA

a Neutrophils were incubated with fluorescence-conjugated mAbs to LFA-1 or PSGL-1 and analyzed by FACScan flow cytometry. LFA-1 receptor number was quantified by comparing the binding of neutrophils to LFA-1-FITC with the binding of receptor-coated microbeads with known binding site densities [61]. PSGL-1 receptor number was determined in a similar fashion [60].

b *Pon* and *Kon* are intended to map to aspects of the same in vitro phenomena. However, there is no direct mapping between these parameters because the parent models belong to fundamentally different classes.

**Table 5:** Experimental values for the blood-perfused micro-flow chamber and the cremaster muscle venule experiments and the corresponding ISWBC2 parameter values used for the two simulated experimental conditions.

Experiment	Experimental Parameter	Experimental Value	Ref	ISWBC Parameter	ISWBC Value
**I. Ex Vivo Blood Perfused Micro-flow chamber**					
	P-Selectin	18 µg/mL	[13]	pselectin	15±5
	ICAM-1	15 µg/mL	[13]	icam1	25±5
	CXCL1	15 µg/mL	[13]	cxcl1	1
	Shear	2.5 dyn/cm^2^	[13]	*RearForce*	0.6
**II. In Vivo Cremaster Muscle Venule**					
	P-Selectin^a^	1.02×10^7^ molecules/cm^2^	[66]	pselectin	12±5
	ICAM-1^a^	8.90×10^7^ molecules/cm^2^	[66]	icam1	6±3
	CXCL1	5 µg/mL	[13]	cxcl1	1
	Shear	1–4 dyn/cm^2^	[52]	*RearForce*	0.6

a Neutrophils were incubated with radiolabelled mAbs to P-Selectin or ICAM-1 and analyzed by laser confocal microscopy. The receptor number is determined by the binding ratio between the fluorescence and immunoglobulin [66].

**Figure 4:**
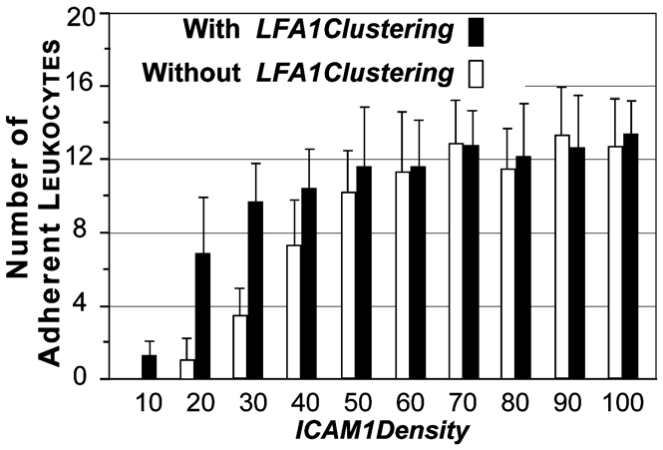
Effect of varying *ICAM1Density* parameter values on leukocyte adhesion. The effect of Leukocyte *ICAM1Density* was varied from 10 to 100 at intervals of 10. Bar heights are ISWBC2 means ± 1 SD (20 populations containing 30 leukocytes each) for the same condition using the parameter values in Tables 4 and 5. Black bars indicate simulation experiments when *LFA1Clustering* = true. White bars indicate simulation experiments when *LFA1Clustering* = false.

We varied the values of *ICAM1Density* (density of icam1) to determine their impact on simulation outcomes. The results in Figure 4 show that at *ICAM1Density* values≥60, disabling clustering (by changing *LFA1ClusteringAllowed* from true to false) did not change adhesion. However, at *ICAM1Density* values≤50, disabling clustering reduced adhesion. The greatest differences were observed at the lower *ICAM1Density* values, indicating that with clustering there was cooperative binding between surfaces. We repeated this set of experiments twice (varying *ICAM1Density* values), but *LFA1GridDensity* (the fraction of all membrane units that contain lfa1grids) values were changed first to 0.1 and then to 0.4. Similar cooperative binding effects were observed at both *LFA1GridDensity* values, but at different ICAM1Density values and with differing magnitudes (Text S1).

The experimental observations above map well to results of wet-lab experimental studies done by Constantin and co-workers. They demonstrated that PI3K inhibition blocked lymphocyte adhesion at low densities of ICAM-1. At high ICAM-1 densities, lymphocytes were able to overcome the requirement for PI3K for efficient adhesion [4].

**Figure 5:**
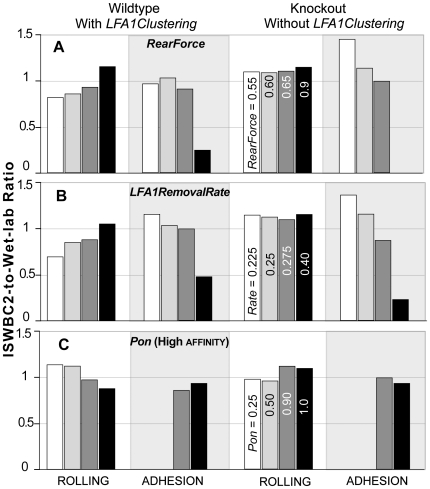
Robustness of ISWBC2s to changes in the *LFA1RemovalRate*, *Pon*, and *RearForce*. Separate sets of in silico experiments, using the leukocyte parameter values in Table 4 and environment parameter values in Table 5, were completed while varying either (A) *RearForce*, (B) *LFA1RemovalRate*, or (C) *Pon*(high affinity lfa1-icam1) as indicated. Bars heights are ratios of ISWBC2-to-wet-lab results of rolling and adhesion data for both WT and KO mice of the type used for the experiments in Figure 3. Comparable adjustments of other parameters caused similar gradual changes in leukocyte rolling and adhesion data. The listed parameter values for rolling under Knockout also apply to the similarly shaded bar for the other three conditions.

Results of sensitivity analysis experiments for changes in *LFA1RemovalRate*, *Pon* (high affinity lfa1-icam1), and *RearForce* are graphed in Figure 5. The simulations were repeated using small (5–10%) and large variations (>50%) in each of these parameters separately, while holding all other parameter values constant. Figure 5 shows the ratio of ISWBC2-to-wet-lab results for rolling and adhesion data from both WT and KO mice for four parameter variations selected to show the trends observed. Small variations in all three parameter values resulted in minimal changes in rolling and adhesion both with and without clustering. The results still matched referent data reasonably well. As was expected, large increases in the *RearForce* (Figure 5A) and *LFA1RemovalRate* (Figure 5B), or a large decrease in *Pon* values (Figure 5C) resulted in a significant decrease in adhesion both with and without clustering. Those results failed to match referent data. Results of these and other robustness explorations (not shown) demonstrated that there are many parameter vectors close to the ones in Table 4 and Table 5 that produce ISWBC2s that validate.

### At Low Densities, lfa1 clustering is Necessary for Sustained leukocyte adhesion

Smith et al. observed individual leukocytes in vivo after injection of CXCL1 into the carotid artery of WT and KO mice to determine its role in adhesion. Events in post-capillary venules were recorded using intravital microscopy. Individual cells were tracked in each vessel starting one minute before and ending one minute after CXCL1 injection. After CXCL1 injection, leukocytes rapidly adhered to the vessel wall and remained attached over time in WT mice. However, in KO mice, leukocytes did not attach or attached transiently.

**Figure 6:**
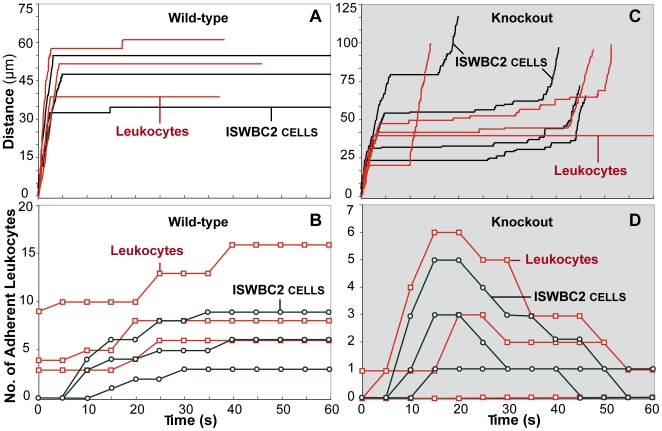
In vivo and in silico results are compared. In vivo conditions (from [13]): mice were either (A, B) WT or (C, D) KO. Red: wet-lab values; black: ISWBC2 values. Wet-lab experiments: post-capillary venules were observed from one minute before to one minute after CXCL1 injection at *t* = 0. Each simulation ran for 600 simulation cycles (equivalent to about 60 seconds). Individual leukocyte trajectories are plotted for (A) WT and (C) KO mice. (B, D) Individual leukocytes were tracked every 5 s, and those that were adherent during that time were counted. Open squares are adherent leukocyte counts for individual venules. Open circles are corresponding adherent leukocyte counts for venules. Thirty leukocytes comprised the population within a venule. Individual leukocytes were tracked every 50 simulation cycles (approximately 5 s), and those that were adherent were counted. ISWBC2: parameter values are those listed in Tables 4 and 5, and leukocytes are in the presence of cxcl1 beginning at t = 0. Lfa1 clustering was either (A, B) enabled or (C, D) disabled.

We simulated similar conditions and observed whether disabling lfa1 clustering would allow the ISWBC2 system to reproduce the observed defect in sustained adhesion. We chose parameter values based on corresponding literature values. When none were available, we searched empirically. Lfa1 clustering was enabled when simulating conditions in WT mice. It was disabled when simulating conditions in KO mice. Figure 6A shows that when lfa1 clustering was enabled, individual leukocytes initiated adhesion within seconds of exposure to cxcl1 and were able to sustain adhesion for the duration of the simulation. Leukocyte population level measurements were also similar to those observed in vivo (Figure 6B).

When lfa1 clustering was disabled to simulate conditions in KO mice, leukocytes exhibited the same transient adhesion observed in vivo (Figure 6C). In the presence of cxcl1 chemokine, leukocytes rolled for a brief period and then initiated adhesion to the surface for a brief interval before again initiating rolling. Figure 6D shows that the similarity in individual leukocyte behaviors translated successfully to population level measurements.

In PI3Kγ KO mice during the above-described experiments, a small portion of leukocytes was still able to sustain adhesion. That observation may implicate an additional mechanism. Using ISWBC2, some leukocytes initiated and maintained adhesion for long intervals. However, none sustained adhesion for the entire duration of the simulation. Additionally, in WT mice, some leukocytes were adherent prior to chemokine injection. The intercept values for the wet-lab data in Figure 6B show that, at the time of injection, adherent leukocytes were already present. That observation may indicate some leukocyte pre-activation. The ISWBC2 system does not include any pre-activation effects. Consequently, we do not observe any adhering leukocytes at the start of a simulation. Nonetheless, the increases in the number of adherent leukocytes after chemokine addition were similar to referent observations.

### Influence of lfa1 clustering on lfa1 and icam1 rebinding Events

**Figure 7:**
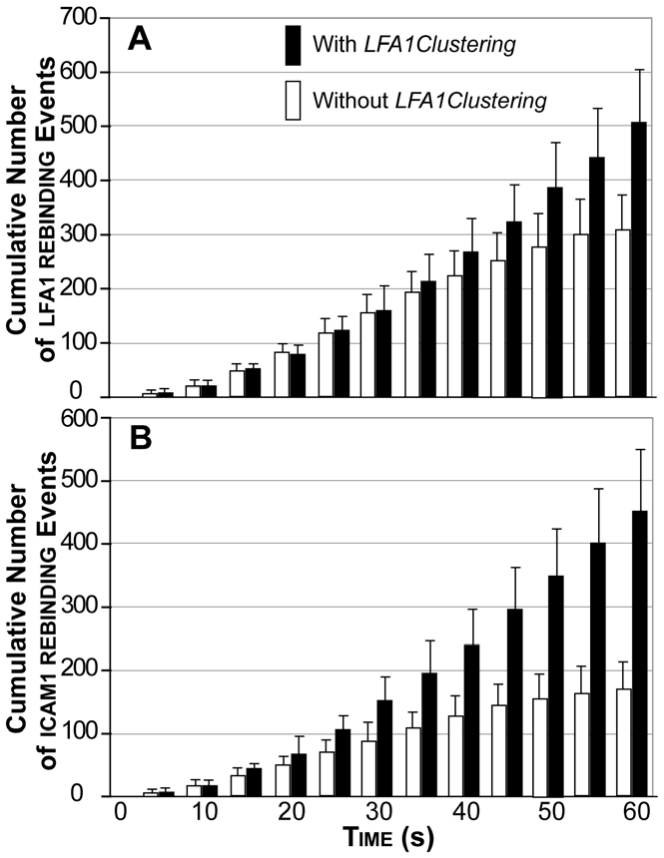
Effect of lfa clustering on lfa1 and icam1 re-binding events. The cumulative number of (A) lfa1 re-binding events and (B) icam1 re-binding events were counted at 50 simulation cycle intervals (5 seconds) for each leukocyte. A lfa1 (or icam1) rebinding event was defined as a bond formation event by a lfa1 (or icam1) object that had already participated in a bond formation event in a previous time step. Averages are plotted for 30 leukocytes per experimental condition (with or without *LFA1Clustering*). Simulation data with *LFA1Clustering* were from leukocytes that sustained adhesion (rolling followed by at least 30 simulation cycles of arrest until the end of the simulation). Simulation data without *LFA1Clustering* were from leukocytes that exhibited initial and transient adhesion (at least 300 simulation cycles of arrest). Without *LFA1Clustering*, the average adhesion time was 39.8±7.2 seconds before the leukocyte initiated rolling again (average time = 43.9±7.1 seconds). Error bars: ± SD.

It has been suggested that integrin clustering may facilitate rebinding events thus enhancing leukocyte adhesion [5],[6]. Rebinding effects are those in which an ICAM-1 that is displaced from one LFA-1 will rapidly bind to a neighboring LFA-1 integrin if it is in sufficiently close proximity. We counted the cumulative number of lfa1 rebinding events (Figure 7A) and icam1 rebinding events (Figure 7B) at 50 simulation cycle intervals (every 5 seconds) for each leukocyte. We defined an lfa1 (or icam1) rebinding event as a bond formation event by an lfa1 (or icam1) that had already participated in a bond formation event during a previous simulation cycle. Averages were taken of 30 leukocytes from the simulations of the in vivo experiments for each experimental condition (*LFA1Clustering* enabled or disabled). All simulation data for the enabled *LFA1Clustering* experimental condition was generated by leukocytes that sustained adhesion (rolling followed by at least 30 simulation cycles of arrest until the end of the simulation), while all simulation data for the disabled *LFA1Clustering* condition was generated by leukocytes that exhibited initial and transient adhesion (at least 300 simulation cycles of arrest; average amount of time leukocytes remained stationary was 39.8±7.2 seconds). The average time that leukocytes detached following transient adhesion was 43.9±7.1 seconds for the disabled *LFA1Clustering* condition.

At simulation times prior to 43.9 seconds, there were no significant differences in lfa1 rebinding events for enabled and disabled *LFA1Clustering* condition (Figure 7A). A significant difference was observed after 43.9 seconds, as expected. leukocytes with *LFA1Clustering* disabled began to roll again after 43.9 seconds allowing different lfa1 objects on the membrane to form new interactions with icam1. Those events caused the number of lfa1 rebinding events to plateau. In contrast, leukocytes with *LFA1Clustering* enabled continued to sustain adhesion enabling the same lfa1 and icam1 objects to interact, as evidenced by the rapidly increasing numbers of lfa1 rebinding events until the end of the simulation.

A different situation was observed with icam1 rebinding. Figure 7B shows a significant difference in icam1 rebinding events when lfa1 clustering is enabled and disabled. As early as 25 seconds, the number of icam1 rebinding events was significantly larger for the lfa1 clustering enabled condition than for the disabled condition. The difference increased throughout the duration of the simulation. These results indicated that when lfa1 was clustered, more lfa1 objects were rebinding to the same icam1 objects than when lfa1 was randomly distributed and non-clustered.

### Comparison of the Effect of icam1 Spatial Arrangements on Sustained adhesion

ICAM-1 has been reported to arrange itself on endothelial cell membrane surfaces in at least four forms: (1) monomeric, (2) dimeric, (3) dimeric that forms linear tetramers upon ligand binding, and (4) preclustered into tetraspanin enriched microdomains. We implemented each of these spatial configurations in order to compare their effect, in combination with lfa1 clustering, on leukocyte's ability to sustain adhesion. We observed the consequences over a range of *LFA1GridDensity* and *ICAM1Density* values. For each icam1 configuration, *LFA1Clustering* parameter setting, *LFA1GridDensity* value, and *ICAM1Density* value, we performed 450 leukocyte simulations within lfa1 and icam1 density ranges that showed the greatest influence of lfa1 clustering. We then calculated the percentage of leukocytes that were able to initiate and sustain adhesion for the duration of the simulation.

**Figure 8:**
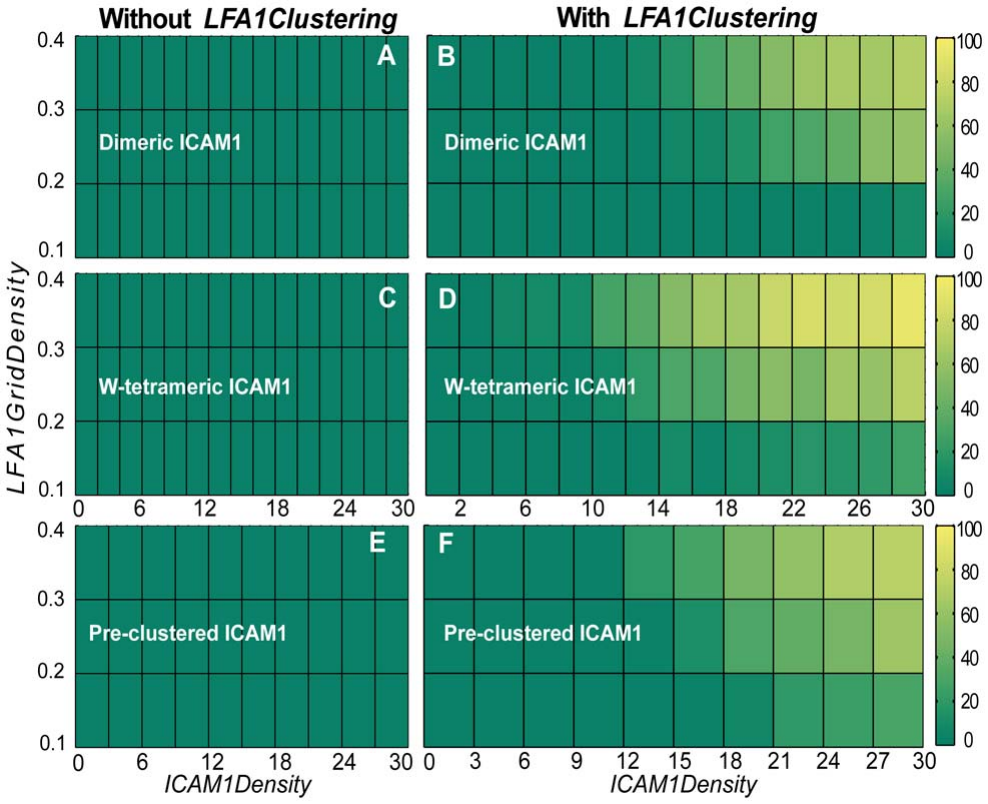
The effect on sustained adhesion of different hypothesized icam1 configurations. The percentage of leukocytes that sustained adhesion (color scale at right) were calculated for varying *LFA1GridDensity* and *ICAM1Density* values using the different hypothesized icam1 configurations discussed in the text. Lfa1 clustering was either disabled (left: A, C, E) or enabled (right: B, D, F). Icam1 was (A, B) dimeric, (C, D) dimeric and allowed to form linear tetramers upon ligand-binding, or (E, F) was preformed into nanoclusters. No sustained adhesion occurred when icam1 was monomeric (not shown).

There was no difference in sustained adhesion between simulations when lfa1 clustering was either enabled or disabled when icam1 existed in a monomeric configuration (not shown). That result was expected because the mechanism is initiated only after a multimeric bond is formed between multiple lfa1 objects and multiple icam1 objects. If icam1 is monomeric, the chance of the lfa1 clustering mechanism being initiated is very small. In contrast, the results in Figure 8 show that, for all multimeric configurations tested, significant differences in sustained adhesion were observed when lfa1 clustering was either enabled or disabled. Whether icam1 existed as a dimer, a dimer that formed into linear tetramers upon ligand-binding, or preclustered produced only slight differences in the percentage of leukocytes that were able to sustain adhesion.

## Discussion

### Achievements

We constructed and validated models of leukocyte rolling, activation, and adhesion. We then experimented on them to test the plausibility of mechanistic hypotheses of how molecular components may interact to cause leukocyte behaviors at the cell and population level. We began with significant leukocyte rolling and adhesion data from ex vivo flow chamber and in vivo mouse cremaster muscle experiments, in which mice lacking functional PI3Kγ exhibited defects in adhesion and sustained adhesion in comparison to leukocytes from WT mice. Smith et al. hypothesized that the adhesion defects were a result of an inability of LFA-1 to redistribute and cluster on the leukocyte membrane in the PI3Kγ KO mice [13].

To challenge that hypothesis, software objects were constructed and assembled according to the mechanistic design in Figure 1 using the operating logic in Text S1. The resulting ISWBC2 system was iteratively refined until validation was achieved across multiple experimental conditions and attributes. LFA1 objects were designed to cluster upon leukocyte activation and post-ligand binding to multimeric icam1. During execution, leukocytes exhibited behaviors indistinguishable from leukocytes observed in both the ex vivo (Figures 3 and 5) and in vivo (Figure 6) experiments using WT mice. More importantly, inhibiting this mechanism allowed leukocyte behaviors to mimic the adhesion defects observed both ex vivo and in vivo in KO mice. Thus, ISWBC2 simulations provide a tested theory about the mechanistic events that may be occurring in both WT and KO mice. At higher lfa1 and icam1 densities, enabling lfa1 clustering did not improve adhesion. However, at low densities, enabling clustering led to cooperativity at the level of the leukocyte-surface zone of contact, and that increased adhesion. Analysis of rebinding events (Figure 7) showed that at later but not earlier times, enabling lfa1 clustering allowed for an increase in lfa1 rebinding events in comparison to when lfa1 did not cluster. Clustering enabled increased icam1 rebinding events at early times. Given the multi-attribute validation evidence, it is reasonable to claim that ISWBC2 mechanisms have mouse counterparts.

Though still relatively simple, we have demonstrated that ISWBC2s can achieve a substantial list of targeted attributes under a variety of experimental conditions (Table 1A, B). Traditional inductive, equation-based models typically focus on just one or a few different experimental conditions. This observation motivates commentary about the differences between traditional, inductive, equation based models and synthetic, relationally grounded analogues like ISWBC2s. The issues are discussed in detail in [11]. Grounding is defined as the units, dimensions, and/or objects to which a variable or model constituent refers [11]. In models grounded to metric spaces, parameters serve mostly to shift model behavior within a smooth region of the output metric space. In relationally grounded models, like ISWBC2s, in addition to that function, parameters also serve to shift model behavior discontinuously (even abruptly) into an entirely different region of behavior space: they change the analogue's dynamic phenotype. In metrically grounded models, the character of the model is bounded, whereas with relational grounding, model character can change completely with a change in parameters. In the former case, parameters describe one, particular (though abstract) model type. In the latter case, parameters describe families of different yet related models. The ISWBC2s functioning in various experimental conditions are examples of the latter. Relational grounding enables flexible, adaptable analogues, but requires a separate analogue-to-referent mapping model.

The process of discovering a normal ISWBC2 that eventually achieved sustained adhesion typical of WT mouse counterparts, and the process of subsequently discovering modifications that eventually showed defective adhesion indistinguishable from that observed in PI3Kγ KO mice, was the same. It followed the iterative refinement protocol. Each ISWBC2 structure and parameterization was a hypothesis: upon execution, as a consequence of combined micro-mechanisms, measures of leukocyte rolling and adhesion will mimic referent data. Execution and measurement provides data that either support or falsify the hypothesis. Early during iterative refinement, all mechanistic hypotheses were falsified: they failed to achieve the prespecified target attributes. Why one was falsified was often somewhat surprising, reflecting uncertainties about the actual referents' underlying mechanisms. At times it reflected incorrect ideas about how micromechanisms influence ISWBC2 behaviors. The many cycles of iterative refinement that followed required and exercised abductive reasoning, which is essential to achieving new scientific insight [39],[40]. A failure of an early ISWBC2 to achieve one or more prespecified attributes taught us something about those ISWBC2s and improved insight into the referent systems. Failure forced us to think more deeply about plausible mechanistic details, and that in turn forced us to think differently about leukocytes and the process of rolling, activation, and adhesion.

Previous studies by Constantin et al. provided evidence of PI3K mediated LFA-1 clustering in immobilized lymphocytes treated with chemokines [4]. Chemokines triggered a rapid increase in LFA-1 affinity and stimulated LFA-1 movement into clusters and large polar patches. Inhibition of PI3K activity blocked LFA-1 mobility but not LFA-1 affinity changes, and that prevented lymphocytes from adhering to low densities of immobilized ICAM-1. At high densities of immobilized ICAM-1, inhibiting PI3K activity had no effect on lymphocyte adhesion. Because the cell type was different, those observations were not among those originally targeted (Table 1). Nevertheless, the validated ISWBC2 that gave the results in Figure 4 correctly predicted those results. Whether this same mechanism is operative and influential in murine neutrophils remains to be determined. However, it is noteworthy that our simulation results are consistent. In ISWBC2 experiments, lfa clustering was important at low icam1 densities, but no differences in adhesion were observed at high icam1 densities (Figure 4).

Lum et al. used sophisticated in vitro methods to study murine neutrophils after stimulation with IL-8 chemokine [3]. They correlated the dynamics of adhesion with the increased expression of high affinity LFA-1 and membrane redistribution. Using fluorescence microscopy, they observed redistribution of high affinity LFA-1 into small punctate submicron clusters and large polar caps several µm^2^ in area within 30 s of IL-8 stimulation. Within 2 min of chemokine stimulation, the polar caps dissipated into numerous smaller clusters. By 10 min, practically all clusters had dispersed and the number of active LFA-1 was observed to have dropped by ∼50%. Inhibition of PI3K activity by treatment with wortmannin did not affect the expression of high affinity LFA-1, but significantly inhibited the amount of LFA-1 clustering and formation of polar caps. Treatment with wortmannin after IL-8 stimulation also significantly decreased the amount of adhesion to fluorescent microbeads coated with ICAM-1 in a flow cytometric based assay. They also used a parallel plate flow chamber coated with an ICAM-1 monolayer to observe the strength and stability of neutrophil adhesion over time. Interestingly, the transience of LFA-1 cluster formation and number of active LFA-1 on the membrane correlated with a reversibility of firm adhesion observed in the flow chamber.

We did not target any of the preceding results. One can question whether the observed behaviors from such in vitro murine neutrophils studies are relevant to those of native cells in the circulation under physiologic conditions [41], such as those used in the ex vivo and in vivo experiments that we targeted. It is recognized that procedures for isolating neutrophils to be studied in vitro can be inefficient and time-consuming. Previous reports have shown that neutrophils become unintentionally modified or activated because of the large number of steps required during isolation [42]–[44]. For example, in vitro isolated and stained neutrophils do not show normal rolling behavior when injected back in mice [38]. Use of the auto-perfused ex vivo flow chamber system allows one to bypass these cell isolation procedures.

### Other Models of Leukocyte Rolling and Adhesion

Bailey et al. constructed a multi-cell, tissue-level, agent-oriented analogue of human adipose-derived stromal cell trafficking through a microvasculature structure within skeletal muscle following acute ischemia [45]. A goal was to identify potential bottlenecks that may limit the efficiency of administered therapeutic cells being recruited into the site of ischemic injury after intravenous injection. They used confocal microscopy images to manually construct an image of the morphology of a characteristic microvascular network. Endothelial cells lining the vessel surface, tissue resident macrophages, circulating monocytes, and therapeutic stem cells were individual agents. The agent-oriented model was coupled with a network blood flow analysis program that calculated blood pressure, flow velocities, and shear stresses throughout the microvascular network.

Each endothelial cell, monocyte, and counterpart to a human, adipose-derived, stromal cell (hasc) could be either positive or negative in expression of each of several adhesion molecules. Similarly, each endothelial cell, monocyte, and tissue resident macrophage could be either positive or negative for secretion of each of the chemokines and cytokines. Whether a circulating monocyte or hasc rolled or adhered depended on whether they experienced a specified combination of adhesion molecule states and chemokine secretion states from a nearby endothelial cell, and whether they experienced a wall shear stress below a certain threshold level. If the cell adhered for more than a specified number of simulation cycles, it transmigrated into the tissue space.

They observed that introduction of an additional adhesion molecule, with properties similar to PSGL-1, enabled the model to more closely mimic in vivo experimental results. They showed that small fractions of hASC's are able to roll on P-selectin even though they do not express PSGL-1. They proposed that the additional adhesion molecule might map to the cellular adhesion molecule CD24. This new knowledge gained reinforces the merit of investigating the complex mechanisms mediating leukocyte adhesion using a synthetic modeling and simulation approach.

While ISWBC2s were constructed using similar methods and components, there are notable differences. The Bailey et al. model focused on leukocyte trafficking events at the tissue level. They explicitly represent in silico counterparts of differing blood pressures, flow velocities, and shear stresses throughout a microvascular network. Leukocytes rolling and adhering on a substrate coated surface within an ISWBC2 system is a small aspect of leukocyte trafficking through microvascular tissue. ISWBC2s focus more on the molecular and cellular level interactions, and have concrete counterparts to the molecular interactions between leukocyte and endothelial cell adhesion molecules.

The discrete-time Adhesive Dynamics (AD) simulations by Hammer and co-workers are the most developed models of leukocyte rolling and adhesion to date [46]–[49]. In their models, leukocytes are idealized as solid spheres with extensible cylindrical protrusions, to represent microvilli, with receptors located at the tips. Using a Monte Carlo algorithm for the determination of receptor-ligand interactions, they have successfully produced a jerky stop-and-go pattern similar to that observed for rolling leukocytes. Their simulations have also allowed them to explore the molecular properties of adhesion molecules, such as reaction rates and bond elasticity, and how these properties may relate to macroscopic behavior such as rolling and adhesion [47],[48].

At each time step in the Adhesive Dynamics simulation, positions of bonds on the spherical particle are tracked enabling the authors to calculate the forces that each bond experiences. The net force and torque acting on the cell from bonds, fluid shear, steric repulsion, and gravity are calculated assuming the cell is a solid sphere. The position of the cell is then determined for each time step from the net force and torque on the cell using a hydrodynamic mobility function for a sphere near a plane wall in a viscous fluid.

In a recent version of their model, they have simulated the transition from rolling to adhesion upon detection of the IL-8 chemokine [49]. To represent the local G-protein intracellular signaling events, they use a 1D lattice of 1000 units on which a small set of intracellular signaling molecules can diffuse and interact. The bottom end of the lattice represents the microvilli tip. In their model, detection of IL-8 by CXCR1 on a microvilli tip initiates the dissociation of G-protein into two subunits, α and βγ, which can then diffuse along the 1D lattice. Effector molecules become activated when bound to the βγ subunit. In turn, the activated effector molecule can then diffuse and bind to the intracellular portion of LFA-1 at the bottom end of the lattice, converting it into a high affinity integrin.

They observed a progressive activation of the integrins as cells rolled and interacted with chemokines, leading to a deceleration of the leukocyte before firm adhesion. The slowing of the leukocyte in their model was on a timescale similar to leukocytes from in vitro experiments. In addition, they were able to observe a chemokine density-dependent effect on adhesion time similar to that observed in vitro.

The ISWBC2 differs in many aspects from this recent version of the AD model, which are briefly discussed below. A more detailed comparison of the ISWBCs with the AD models and with other models of leukocyte motility and adhesion can be found in the Appraisal of Model Specifications section within Text S1 and in [12].

In the above AD model, LFA-1 is found at the tips of microvilli. Studies have shown that LFA-1 exists on the cell body surface hidden between the leukocyte microvilli [29],[30]. Therefore, in our ISWBC2 we have specified simply that lfa1 objects are located on sparse regions on the membrane surface. The fraction of membrane units containing lfa1grids and lfa1 objects was determined by the parameter *LFA1GridDensity*.

The most recent version of the AD simulations also included a more explicit representation of the signaling events initiated upon chemokine detection. With the ISWBC2, it was not our objective to model the signaling network in detail, and therefore it was not listed as a currently targeted phenotypic attribute in Table 1. Our current goal was to test the hypothesized molecular mechanisms at the cell interface of the leukocyte and endothelial substrate surface. However, we have shown previously that synthetic models like ISWBCs can be easily refined to become increasingly realistic in terms of both components and behaviors [12]. As needed, any of the abstract, low-resolution components can be replaced with more realistic, higher resolution composite objects composed of components that map to more detailed biological counterparts. The current ISWBC2 represents the hypothesized mechanisms and processes at a level of detail and resolution that is just sufficient to simulate the currently targeted attributes listed in Table 1.

### Future Directions

The ISWBC2 system represents significant progress towards a larger goal of discovering and validating concrete plausible mechanistic details of cell-cell interactions, in the face of considerable uncertainty, by building computational devices comprised of instantiated mechanisms. ISWBC2s are capable of mimicking only a portion (Table 1) of a long list of targeted phenotypic attributes. However, we have shown here and previously that our iterative refinement method provides a means to concatenate in a dynamic fashion new knowledge of leukocyte rolling and adhesion into our ISWBC systems as it becomes available, without having to reengineer the whole system, and without having to compromise already validated features and behaviors [12]. The expectation is that ISWBCs can be iteratively refined to become increasingly realistic in terms of components, mechanisms, and behaviors. The greater the similarity, the more useful the analogue will become as an observable expression of coalesced, relevant leukocyte knowledge. Future targeted attributes should include those associated with abnormal disease-associated leukocyte adhesion, and predicting the consequences of interventions.

## Supporting Information

Text S1 Supplementary Text: Decisional Processes for LFA1, PSELECTIN, MEMBRANE UNIT, LEUKOCYTE MEMBRANE, and ICAM1; BOND Formation and Dissociation; Diffusion; Effect of varying ICAM1Density and LFA1Density on LEUKOCYTE ADHESION; and Appraisal of Model Specifications
(1.44 MB PDF)
