# Supplementary material for: Identifying the Rules of Engagement Enabling Leukocyte Rolling, Activation, and Adhesion
Source: PLoS Comput Biol. 2010 Feb 19;6(2):e1000681. doi: 10.1371/journal.pcbi.1000681 (PMC2824748; doi:10.1371/journal.pcbi.1000681)
Supplement: Text S1 — Supplementary Text: Decisional Processes for LFA1, PSELECTIN, MEMBRANE UNIT, LEUKOCYTE MEMBRANE, and ICAM1; BOND Formation and Dissociation; Diffusion; Effect of varying ICAM1Density and LFA1Density on LEUKOCYTE ADHESION; and Appraisal of Model Specifications (1.44 MB PDF) [file pcbi.1000681.s001.pdf]

Identifying the Rules of Engagement Enabling Leukocyte Rolling, Activation, and Adhesion –

SUPPLEMENTARY INFORMATION

J. Tang and C.A. Hunt

1 Decisional Processes for LFA1, PSELECTIN, MEMBRANE UNIT, LEUKOCYTE MEMBRANE, and ICAM1

Figure S1 was referred in the *Behaviors* subsection under Methods.

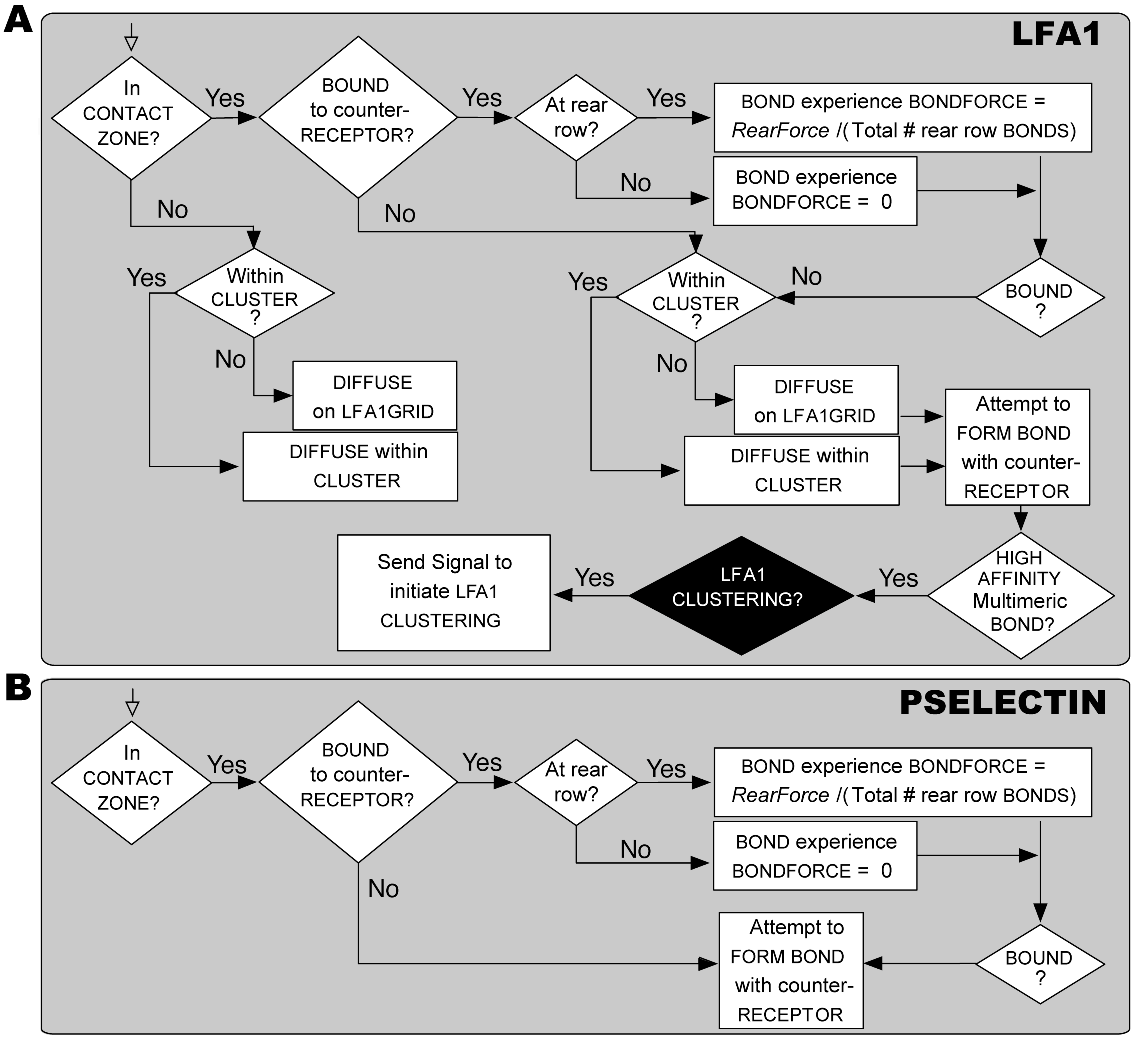

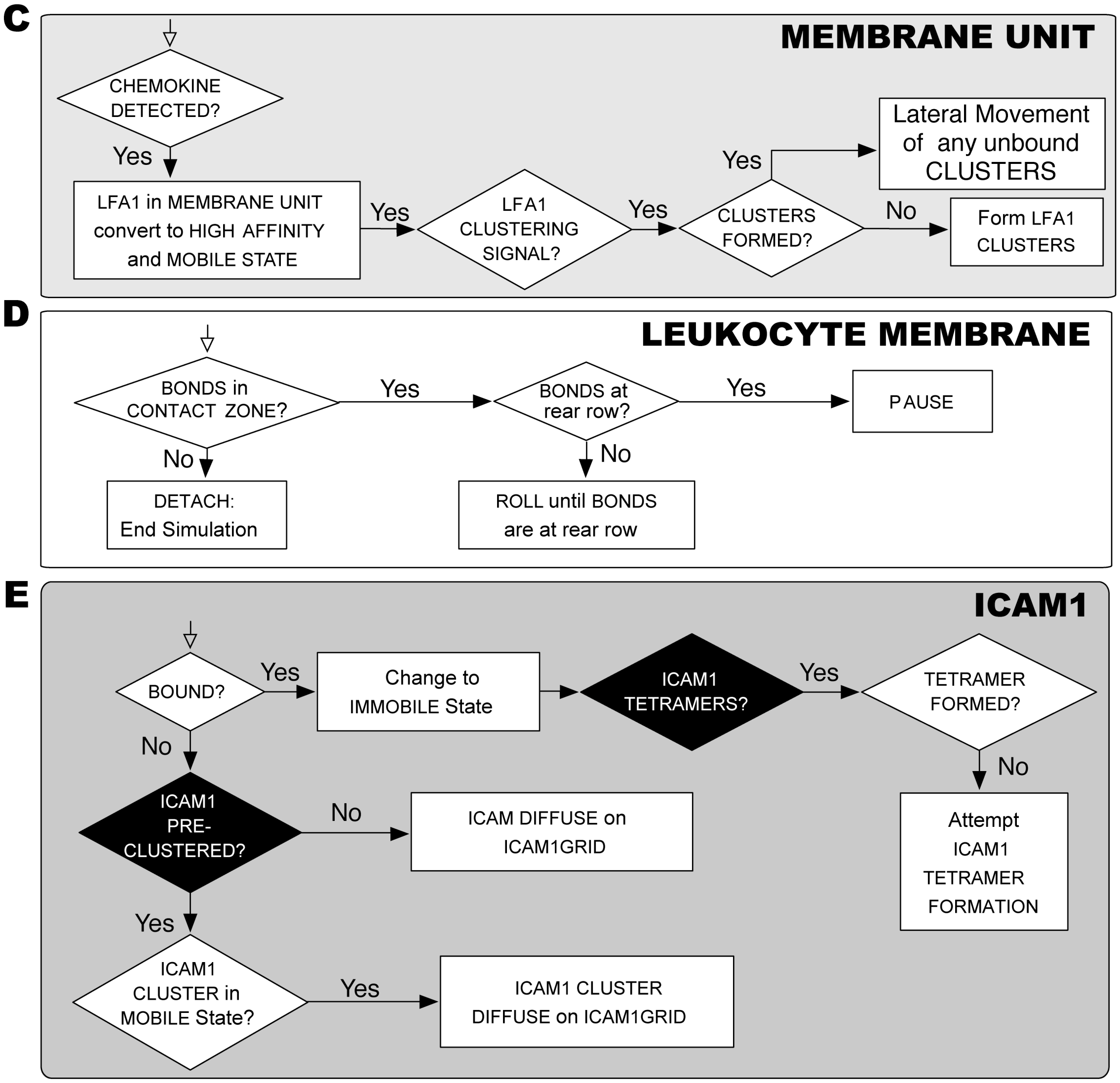

Figure S1. Decisional processes for LFA1, PSELECTIN, MEMBRANE UNIT, LEUKOCYTE MEMBRANE, AND ICAM1. Sketched is the decisional process for the (**A**) LFA1, (**B**) PSELECTIN, (**C**) LEUKOCYTE MEMBRANE UNIT, (**D**) LEUKOCYTE MEMBRANE, and (**E**) ICAM1 during a simulation cycle. Black diamonds are Boolean variables that determine which behaviors are allowed during a simulation experiment, allowing us to manipulate them individually, or in combination, and to observe the overall emergent effect on LEUKOCYTES and LEUKOCYTE populations. White arrows indicate the starting point for each agent in their decisional process.

2 BOND Formation and Dissociation

BOND formation, dissociation, and Figure S2 are mentioned in the *RECEPTOR Behaviors* subsection under Methods. BOND formation events occur when a RECEPTOR in a MEMBRANE UNIT encounters its counter-RECEPTOR in an overlapping SURFACE UNIT in the CONTACT ZONE. Probability of a BOND formation event is determined by the parameter P*on* for that RECEPTOR-LIGAND pair. For each potential BOND, the value of parameter *Pon* for that RECEPTOR-LIGAND pair is compared to a randomly generated number from a uniform distribution, U(0,1), to determine if that potential BOND becomes an actual BOND.

The effect of shear on the rear of a leukocyte is represented by the variable *RearForce*. BONDS at the rear experience a *bondforce* that is calculated each simulation cycle by dividing the *RearForce* value by the total number of BONDS in the rear row of the CONTACT ZONE. BONDS within the rest of the CONTACT ZONE experience no *bondforce*. Drawing from in vitro data, we have assumed simple linear relationships between *bondforce* and the probability of BOND dissociation for each of the receptor-ligand pairs in our model (Fig. S1). It is calculated as (probability of dissociation) = b0 + (*bondforce*) x b1, where b1 and b0 are the slope and intercept, respectively, of the line segment associated with a specific *bondforce*. Each type of simulated adhesion molecule pair uses a unique set of b0 and b1 values.

Park et al. calculated PSGL-1/P-selectin dissociation rates as a function of force experienced by the bond by observing PSGL-1 covered microbeads rolling on P-selectin substrate in a parallel plate flow chamber. For the range of dissociation rate constants relevant to this report (*Koff* values < 10/s), the in vitro data, as shown in Fig. S2B, is close to linear (the values in Fig. S2B were calculated from the reported, best-fit values [1]). The unstressed dissociation constant, K^0off, for PSGL-1/P-selectin bonds was calculated to be 1.6/s.

Zhang et al. used atomic force spectroscopy to determine the strength of the LFA-1/ICAM-1 complex [2], and single-molecule dynamic force spectroscopy to investigate the strength of the VLA-4/VCAM-1 complex [3]. The experimental conditions for both studies were not the same as ex vivo or in vivo. Nevertheless, the relative behaviors they observed for LFA-1/ICAM-1 and VLA-4/VCAM-1 complexes are expected to be similar to that in the flow chamber. Measurements revealed two activation barriers in the dissociation of the LFA-1/ICAM-1 complex. The outer barrier is the rate-limiting step in the dissociation of the unstressed complex. The K^0off values for the outer energy barriers for the low- and high-affinity LFA-1/ICAM-1 complexes were 4/s and 0.17/s, respectively. The dissociation of the VLA-4/VCAM-1 complex was also determined to involve overcoming two activation energy barriers. Under pulling forces < ~50 pN, dissociation rates were governed principally by the properties of the outer activation energy barrier. The K^0off values for the outer energy barriers for the low- and high-affinity complexes were 1.4/s and 0.0035/s, respectively. The high affinity LFA-1/ICAM-1, low affinity LFA-1/ICAM-1, and high affinity VLA-4/VCAM-1 data in Fig. S1B were converted from the reported semi-log plots [2], [3]. The force dependence of dissociation rate for low affinity VLA-4/VCAM-1 was not reported. We assumed that it is similar to PSGL-1/P-selectin, and represented it so in Fig. S2A.

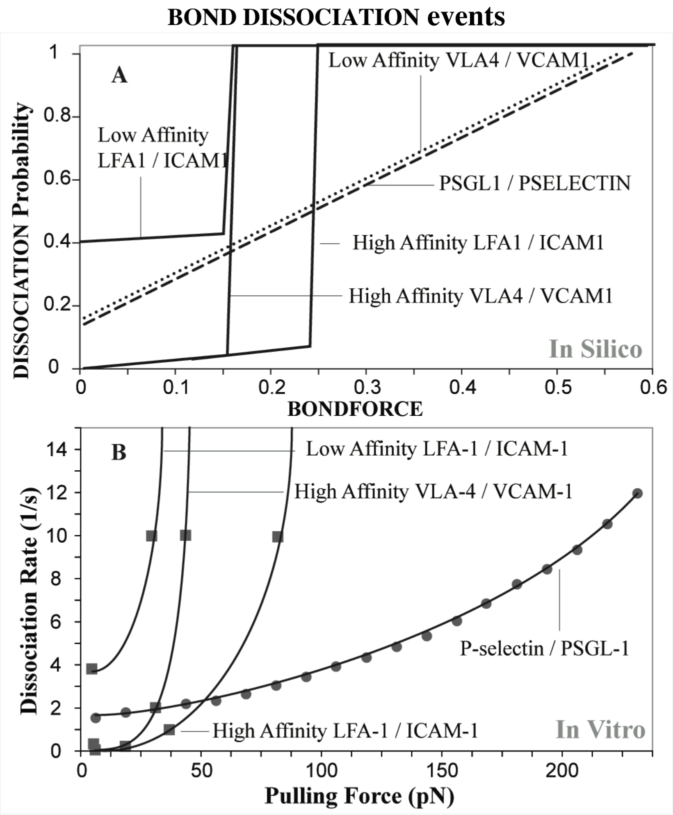

Figure S2. FORCE dependence on BOND DISSOCIATION probability and force dependence on bond dissociation rates. (**A**) Shown is the relationship between *bondforce* and probability of BOND DISSOCIATION for each of the four LIGAND pairs included in the ISWBC. The effects of shear on the ligand-ligand bonds that form at the rear of the leukocyte are simulated using *bondforce*. BONDS within the rear row of the CONTACT ZONE experience a *bondforce* that is calculated by dividing the *RearForce*, a unitless parameter representing the effects of shear, by the total number of BONDS within the rear row. During a simulation cycle, each MEMBRANE UNIT in the rear row uses the current value of *bondforce* and the graphed relationship to calculate a probability that each BOND will be broken during that cycle. All BONDS elsewhere within the CONTACT ZONE experience a *bondforce* value of 0. UNSTRESSED (*bondforce* value of 0) DISSOCIATION probabilities for PSGL1/PSELECTIN, LOW-AFFINITY VLA4/VCAM1, HIGH-AFFINITY VLA4/VCAM1, LOW-AFFINITY LFA1/ICAM1, and HIGH-AFFINITY LFA1/ICAM1 were specified to be 0.14, 0.16, and 0.0035, 0.4, and 0.017 respectively. (**B**) The in vitro force dependence of dissociation rates for P-selectin/PSGL-1 bonds (as reported in [1]) and the high affinity VLA-4/VCAM-1 bonds (as reported in [3]) are plotted for comparison to the analogue relationships in **A**. The plotted values were taken from the fitted in vitro data: see Methods for details. The relationships in **A** are analogues of these experimentally determined relationships and are not meant to either match or fit that data. The dissociation rates of the PSGL-1/P-selectin bonds as a function of force were determined by experiments using PSGL-1-coated microbeads rolling on a P-selectin substrate in a parallel plate flow chamber [1]. The dissociation rates for the LFA-1/ICAM-1 complex were calculated from data obtained using atomic force spectroscopy [2]. The dissociation rates for the VLA-4/VCAM-1 complex were calculated from data obtained using single-molecule dynamic force spectroscopy [3]. The force dependence of dissociation rates for low affinity VLA-4/VCAM-1 data was not reported. We assumed that it is similar to PSGL-1/P-selectin relationship in **A**.

3 Diffusion

LFA1 DIFFUSION and TABLE S1 are mentioned in the *RECEPTOR Behaviors* subsection under Methods. LFA1 lateral mobility parameters were determined such that they have similar relative diffusive properties as observed in vitro. At each time step, each LFA1 object is allowed to randomly move to any of the 6 neighboring hexagonal grid sections on the LFA1GRID, for a specific number of times determined by the parameter *LFA1MoveNum*. We explored several parameter values for *LFA1MoveNum* and calculated the diffusion coefficients using Eq 1. $<r^2>$ is the MSD, t is the time interval, D is the time-dependent diffusion coefficient, and the α coefficient classifies the mode of anomalous diffusion.

$$< r^2> = 4Dt^{\alpha} \qquad \text{[Eq. 1]}$$

Cairo et al. used single-particle tracking to determine the diffusion coefficients of LFA-1 on peripheral blood lymphocytes prior to and after activation with phorbol-12-myristate-13-acetate [4]. They observed in all cells before and after activation the existence of two subpopulations of LFA-1, immobile and mobile, distinguished by their diffusive properties. Diffusion coefficients have not been determined for neutrophils. We assumed neutrophil LFA-1 had similar diffusive properties. We first sought *LFA1MoveNum* parameter values that would allow our LFA1 objects to exhibit similar diffusive properties as those in the mobile and immobile subpopulations. In their calculations of diffusion coefficients, Cairo et al. observed values of α that were consistent with Brownian diffusion ($0.7 < \alpha < 1.2$), and therefore we calculated D values using a α value of 1. We determined that a *LFA1MoveNum* of 10 gave similar diffusive properties to those of the immobile subpopulation, while a *LFA1MoveNum* of 120 yielded similar diffusive properties to the mobile subpopulation (Table S1).

Table S1.

	In Vitro		In Silico	
	Diffusion Coefficient (Untreated) [10^{-10} cm^{2}s^{-1}]	Diffusion Coefficient (PMA Treated) [10^{-10} cm^{2}s^{-1}]	*LFA1MoveNum*	Diffusion Coefficient (maps to) [10^{-10} cm^{2}s^{-1}]
Immobile LFA-1	0.14 ± 0.73	0.11 ± 0.71	10	0.19 ± 0.05
Mobile LFA-1	3.8 ± 1.9	1.3 ± 1.0	120	2.1 ± 0.3

4 Effect of varying *ICAM1Density* and *LFA1GridDensity* on LEUKOCYTE ADHESION

This section and Figure S3 are mentioned in the *LFA1 CLUSTERING and LEUKOCYTE ADHESION* subsection under Results. We repeated the experiments aimed at identifying the effect of *ICAM1Density* on LEUKOCYTE ADHESION, but changed the *LFA1GridDensity* parameter value (the fraction of all MEMBRANE UNITS that contain LFA1GRIDS) from 0.2 to 0.1. When *LFA1GridDensity* was decreased to 0.1 (Figure. S3A), the *ICAM1Density* below which the influence of CLUSTERING became significant shifted from 50 to 70. The magnitude of the clustering effect increased at lower *ICAM1Density* values. On the other hand, when *LFA1GridDensity* was increased, for example from 0.2 to 0.4 (Fig. S3B), the *ICAM1Density* below which the influence of CLUSTERING became significant shifted from 50 to 30. That trend

continued at larger *LFAGridDensity* values. However, in both cases, cooperative binding effects were still observed.

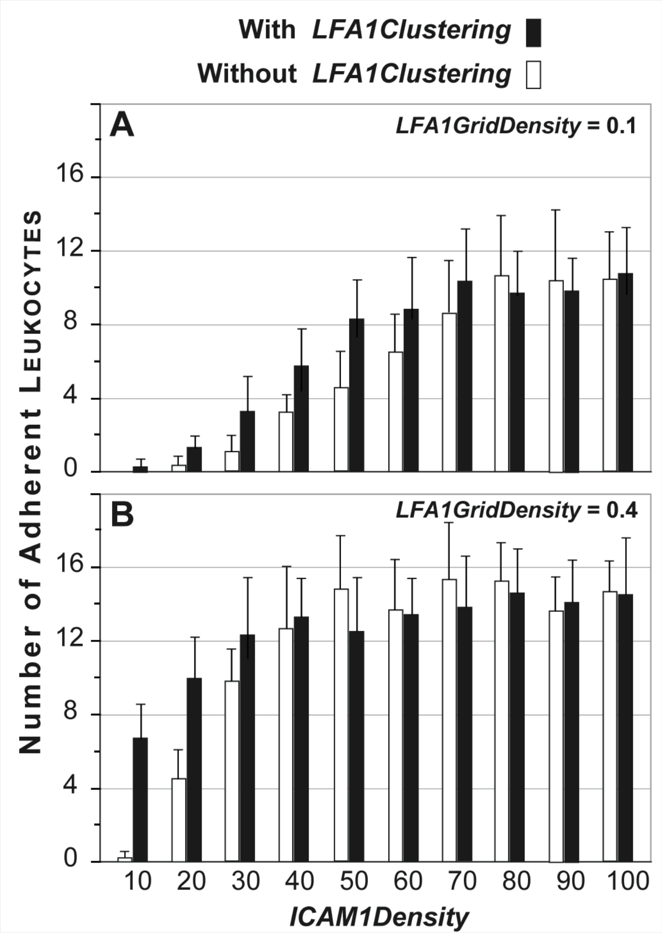

Figure S3. Effect of varying *ICAM1Density* and *LFA1GridDensity* parameter values on LEUKOCYTE ADHESION. The effect of Leukocyte *ICAM1Density* was varied from 10 to 100 at intervals of 10. LFA1GridDensity was set to (A) 0.1 or (B) 0.4. Bar heights are ISWBC2 means ± 1 SD (20 populations containing 30 leukocytes each) for the same condition using the parameter values in Tables 4 and 5. Black bars indicate simulation experiments when *LFA1Clustering* = true. White bars indicate simulation experiments when *LFA1Clustering* = false. Cooperative binding effects were observed both at *LFA1GridDensity* values of (A) 0.1 and (B) 0.4, but at different ranges of ICAM1Density values and with differing magnitudes.

5 Appraisal of Model Specifications

This section was cited in the *Other Models of Leukocyte Rolling and Adhesion* subsection under Discussion.

Following the parsimony guideline, we strove to assemble a system of interacting components that would, at the margin, be complicated enough to exhibit multiple, different targeted attributes under different experimental conditions, but not so complicated that a component could be eliminated without significantly degrading behavioral similarities for the full set of targeted attributes. Some ISWBC2 components map to a conflated set of leukocyte features. Some leukocyte features have no ISWBC2 counterparts. The latter does not mean that those features were ignored or assumed unimportant. At each stage of iterative refinement, a goal was to determine the degree of biomimicry (level of biological emulation) that could be accomplished with a few components before concluding that others may be needed.

Both wet-lab experimental systems used whole blood containing red blood cells in addition to leukocytes. It is well known that smaller red blood cells can enhance leukocyte-substratum interactions by pushing the larger leukocytes from the axial flow to the vessel wall [5], [6], [7]. We do not discount the significance of such interactions under some conditions, however we chose specifically to focus on the interactions between an already attached leukocyte and its surface. At the beginning of each simulation, LEUKOCYTES were placed on the surface where interactions were allowed to form (or not). We posited that red blood cell—leukocyte interactions were similar in WT and KO mice, contributing equally to differences in wet-lab results, and so could be ignored.

The flow chamber system was perfused by murine blood. The dynamics of leukocyte rolling and adhesion may have been influenced by the pulsatile blood flow. A rheological evaluation of the autoperfused flow chamber system was undertaken and reported stable flow conditions and tolerable changes in blood-flow velocity and wall shear stress. Flow through the chamber was essentially laminar and quasi-steady, as reflected by a Reynolds number less than three and a Womersley number of 0.5 [8]. In vivo, wall shear stress varies along the venular tree as vessel diameter and flow rate change. We started by specifying a constant SHEAR STRESS (REARFORCE) at all locations and times within in silico experiments. Our protocol called for retaining that parsimonious specification until falsification of a particular ISWBC2 required adopting a more fine-grained specification, which it did not. Varying REARFORCE can be implemented, as done earlier [9], when that is needed. Adhering to the parsimony guideline within the iterative refinement protocol has proven an effective tool in preventing ISWBC2s from becoming unnecessarily complicated.

With their Adhesive Dynamics (AD) model of leukocyte rolling and adhesion, King and Hammer demonstrated that only bonds at the trailing edge of the contact zone were under stress and that increases in instantaneous velocity correlated with breakage of trailing edge bonds [10]. They state that it is reasonable to simplify the force and torque balances such that only bonds at the trailing edge of the contact zone experience force [11]. Therefore, we have implemented the model specification that only bonds at the rear of the leukocyte experiences force, and that this is shared equally among all bonds at the rear.

Our approach has been focused on simulating a targeted set of system-level properties. It was not our intention to discover ISWBC parameterizations that would yield simulation results that tightly fit specific sets of experimental data. Therefore, we did not, for example, attempt an explicit mapping between the shear force and the amount of force experienced by bonds at the rear of the leukocyte. Leukocytes are notoriously deformable and therefore we elected to avoid a mapping such as the Goldman equation [12], which has been used in the AD models to calculate the amount of force a sphere experiences from an applied shear force by assuming that the sphere is solid. In addition, the mapping from the force on the cell to the force on the rear bonds can be

quite complex as PSGL-1 and VLA-4 are both ligands that are concentrated at the tips of stretchy and heterogeneous microvilli. It should be noted that an estimate of 124.4 ± 26.1 pN per dyn/cm^2 wall shear stress for selectin tethers at the rear of neutrophils has been calculated previously using the Goldman equation [13].

To account for the effect of an applied force on the kinetics of bond dissociation in their AD simulations, Hammer and co-workers employed both the Bell model and Dembo Hookean Spring model (see [14]). The Bell Model predicts the bond dissociation rate as a function of applied force, whereas the Dembo model treats bonds as Hookean springs and relates bond dissociation rate to the length of the stretched bond. Use of a similar fine-grained representation of bond dissociation may have yielded more precise simulation results, but that level of resolution and precision was not needed to meet the simulation objectives in Table 1. We used a simple model to relate the probability of bond dissociation with bond force (Fig. S2A); it was motivated by in vitro experiments by Park et al. [15], and Zhang et al. [16]. We previously determined that this implementation was sufficient [9].

Many molecular level details are believed to impact effective bond formation and breakage within small portions of a leukocyte membrane and the corresponding portion of the surface. Examples include contact irregularities, local dynamics and ligand relocation within the membrane, force history of bond loading, and bond compliance. All of these factors are aggregated and controlled in the ISWBC by an event probability. When explanation of system level behaviors requires a more detailed representation, one or more of these factors can be specifically represented, without compromising the function of other ISWBC components. The probability parameters will remain, but their values and explanation will have changed.

There are many adhesion molecules present on the endothelial surface with varying site densities. Additionally, there is an endothelial surface layer. Studies have shown that the endothelial surface layer may slow plasma flow and may limit the exposure of adhesion molecules [17]. We specified two classes of ENDOTHELIAL ADHESION objects. One maps to P-selectin and any other molecules that behave similarly under the referent wet-lab conditions. The other maps to ICAM-1 and any similarly behaving molecules. We also specified two classes of LEUKOCYTE ADHESION objects. One maps to PSGL-1 and any other molecules that behave similarly under the referent wet-lab conditions. The other maps to LFA-1 and any similarly behaving molecules. We specified one class of CHEMOKINE RECEPTOR objects. It maps to CXCR2 and any other chemokine receptors behaving similarly under the referent wet-lab conditions.

We started by specifying simply that LEUKOCYTES would interact with a layer of randomly distributed RECEPTOR and CHEMOKINE objects. The nature of the actual surface to which those objects are attached was not specified. The RECEPTOR and CHEMOKINE objects map to reactive counterparts that are sufficiently exposed to react. We did not encounter non-matching simulation results that would have triggered revising that specification. Consequently, ISWBC2 systems map equally well to those having engineered and endothelial surfaces.

CXCL1 is not a proinflammatory chemokine. We can therefore posit that ISWBC2 system surfaces map to inactive endothelial cells having a basal expression of adhesion molecules [18]. Again, when it is needed, it is straightforward to add objects that map to endothelial surface features without having to reengineer other features of the ISWB2 system.

References

1. Park EY, Smith MJ, Stropp ES, Snapp KR, DiVietro JA, et al. (2002) Comparison of PSGL-1 microbead and neutrophil rolling: microvillus elongation stabilizes P-selectin bond clusters. Biophys J 82: 1835-1847.

2. Zhang X, Wojcikiewicz E, Moy VT (2002) Force spectroscopy of the leukocyte function-associated antigen-1/intercellular adhesion molecule-1 interaction. Biophysical Journal 83: 2270-2279.

3. Zhang X, Craig SE, Kirby H, Humphries MJ, Moy VT (2004) Molecular basis for the dynamic strength of the integrin a4b1/VCAM-1 interaction. Biophys J 87: 3470-3478.

4. Cairo CW, Mirchev R, Golan DE (2006) Cytoskeletal regulation couples LFA-1 conformational changes to receptor lateral mobility and clustering. Immunity 25: 297-208.

5. Melder RJ, Munn LL, Yamada S, Ohkubo C, Jain RK (1995) Selectin- and integrin-mediated T-lymphocyte rolling and arrest on TNF-activated endothelium: Augmentation by erythrocytes. Biophys J 69: 2131-2138.

6. Firrell JC, Lipowsky HH (1989) Leukocyte margination and deformation in mesenteric venules of rat. Am J Physiol 256: H1667-1674.

7. Schmid-Schonbein GW, Usami S, Skalak R, Chien S (1980) The interaction of leukocytes and erythrocytes in capillary and postcapillary vessels. Microvasc Res 19: 45-70.

8. Smith ML, Sperandio M, Galkina EV, Ley K (2004) Autoperfused mouse flow chamber reveals synergistic neutrophil accumulation through P-selectin and E-selectin. J Leukoc Biol 76: 985-993.

9. Tang J, Ley KF, Hunt CA (2007) Dynamics of in silico leukocyte rolling, activation, and adhesion. BMC Syst Biol 1.

10. King MR, Hammer DA (2001) Multiparticle adhesive dynamics. Interaction between stably rolling cells. Biophys J 81: 799-813.

11. Krasik EF, Hammer DA (2004) A semianalytic model of leukocyte rolling. Biophys J 87: 2919-2930.

12. Goldman AJ, Cox RG, Brenner H (1967) Slow viscous motion of a sphere parallel to a plane wall. II. Chem Eng Sci 22: 635-660.

13. Alon R, Chen S, Puri KD, Finger EB, Springer TA (1997) The kinetics of L-selectin tethers and the mechanics of selectin-mediated rolling. J Cell Biol 138: 1169-1180.

14. Zhu C, Bao G, Wang N (2000) Cell mechanics: mechanical response, cell adhesion, and molecular deformation. Annu Rev Biomed Eng 2: 189-226.

15. Park EY, Smith MJ, Stropp ES, Snapp KR, DiVietro JA, et al. (2002) Comparison of PSGL-1 microbead and neutrophil rolling: microvillus elongation stabilizes P-selectin bond clusters. Biophys J 82: 1835-1847.

16. Zhang X, Craig SE, Kirby H, Humphries MJ, Moy VT (2004) Molecular basis for the dynamic strength of the integrin a4b1/VCAM-1 interaction. Biophys J 87: 3470-3478.

17. Smith ML, Sperandio M, Galkina EV, Ley K (2004) Autoperfused mouse flow chamber reveals synergistic neutrophil accumulation through P-selectin and E-selectin. J Leukoc Biol 76: 985-993.

18. Smith DF, Deem TL, Bruce AC, Reutershan J, Wu D, et al. (2006) Leukocyte phosphoinositide-3 kinase {gamma} is required for chemokine-induced, sustained adhesion under flow in vivo. J Leukoc Biol 80: 1491-1499.
